# Bioactivity Potential of Marine Natural Products from Scleractinia-Associated Microbes and In Silico Anti-SARS-COV-2 Evaluation

**DOI:** 10.3390/md18120645

**Published:** 2020-12-16

**Authors:** Eman Maher Zahran, Amgad Albohy, Amira Khalil, Alyaa Hatem Ibrahim, Heba Ali Ahmed, Ebaa M. El-Hossary, Gerhard Bringmann, Usama Ramadan Abdelmohsen

**Affiliations:** 1 Department of Pharmacognosy, Faculty of Pharmacy, Deraya University, Universities Zone, New Minia City 61111, Egypt; eman.maher@deraya.edu.eg (E.M.Z.); heba.ali@deraya.edu.eg (H.A.A.); 2 Department of Pharmaceutical Chemistry, Faculty of Pharmacy, The British University in Egypt (BUE), El-Sherouk City 11837, Egypt; albohy@ualberta.ca (A.A.); amira.khalil@bue.edu.eg (A.K.); 3 Department of Pharmacognosy, Faculty of Pharmacy, Sohag University, Sohag 82524, Egypt; alyaa_hatem@pharm.sohag.edu.eg; 4 National Centre for Radiation Research & Technology, Egyptian Atomic Energy Authority, Ahmed El-Zomor St. 3, El-Zohoor Dist., Nasr City, Cairo, 11765, Egypt; ebaa.elhossary@eaea.org.eg; 5 Institute of Organic Chemistry, University of Würzburg, Am Hubland, 97074 Würzburg, Germany; 6 Department of Pharmacognosy, Faculty of Pharmacy, Minia University, Minia 61519, Egypt

**Keywords:** Scleractinia, marine bacteria, marine fungi, zooxanthellae, marine natural products, ADME analysis, SARS-CoV-2, molecular docking, RNA-dependent RNA polymerase, methyltransferase

## Abstract

Marine organisms and their associated microbes are rich in diverse chemical leads. With the development of marine biotechnology, a considerable number of research activities are focused on marine bacteria and fungi-derived bioactive compounds. Marine bacteria and fungi are ranked on the top of the hierarchy of all organisms, as they are responsible for producing a wide range of bioactive secondary metabolites with possible pharmaceutical applications. Thus, they have the potential to provide future drugs against challenging diseases, such as cancer, a range of viral diseases, malaria, and inflammation. This review aims at describing the literature on secondary metabolites that have been obtained from Scleractinian-associated organisms including bacteria, fungi, and zooxanthellae, with full coverage of the period from 1982 to 2020, as well as illustrating their biological activities and structure activity relationship (SAR). Moreover, all these compounds were filtered based on ADME analysis to determine their physicochemical properties, and 15 compounds were selected. The selected compounds were virtually investigated for potential inhibition for SARS-CoV-2 targets using molecular docking studies. Promising potential results against SARS-CoV-2 RNA dependent RNA polymerase (RdRp) and methyltransferase (nsp16) are presented.

## 1. Introduction

Marine ecosystems harbor numerous, still unexplored and taxonomically diverse micro- and macro-organisms. These marine organisms have the ability to produce novel compounds, as secondary metabolites, with a wide structural diversity and various important pharmacological potentials [1,2,3,4,5,6]. Among marine organisms, coral reefs are considered to be the biologically most diverse ecosystem on earth. Moreover, it is recognized as an important source of bioactive secondary metabolites [7]. Scleractinia is the order involving stony corals, which are a clade of cnidarians that build a calcium carbonate skeleton. It includes 31 families, about 240 genera and over 1500 species [8,9]. Corals usually harbor vast communities of beneficial microbes [10]. The coral holobiont is composed from the coral animal and its associated microorganisms consisting of bacteria and fungi, complemented by symbiont unicellular organisms known as zooxanthellae [11]. The coral holobiont is a very dynamic ecosystem, where the members vary according to environmental conditions and their daily requirements [12]. Coral-associated microbes are existing in a number of habitats related to corals, including mucus on coral surfaces, intracellular spaces within coral tissues, areas within coral skeletons, and the surrounding seawater [13]. Each of these habitats is believed to harbor different bacterial populations [14]. Despite high microbial diversity, corals have been reported to host species-specific microbial communities for beneficial effects [15,16]. Microbial associations over evolutionary time scales are likely to contribute to genome differentiation in both the host and its associated microbial partners, a process referred to as hologenome evolution [14]. Evidence that metabolic interaction between holobiont members shaped genome evolution may be in the form of genetic systems acquired by the host genome by horizontal gene transfer [17]. Meanwhile it has been proven that the survival of Scleractinia against dramatic environmental changes over time, its adaption to new environmental conditions, in addition to its resistance to coral-specific pathogens is linked to coral-associated microbes [18]. For several decades, marine microorganisms have been drawing research attention, as many new and potent bioactive compounds have been discovered from bacteria and fungi residing on many marine macro-organisms, especially on corals [19,20]. Researchers are still investigating the associations that occur among microbes, their hosts, and the individuals of the community sharing the same habitat. Now, it is becoming evident that these associations involve the sharing of complex chemical signals either they are competitive or mutually advantageous, which may be reflected in the genome composition of the partners eventually leading to the observed variety of bioactive secondary metabolites [21,22].

**Figure 1:**
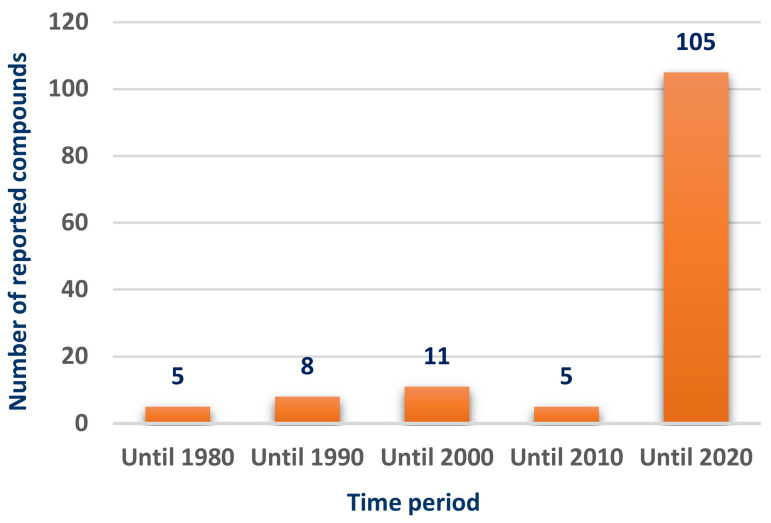
Number of compounds isolated from Scleractinia-associated organisms according to the year of publication.

Scleractinia-associated organisms have been attracting the attention as a potential source of bioactive compounds during the past decade, reflecting the great increase in the number of the isolated compounds over the past ten years (Figure 1).

**Figure 2:**
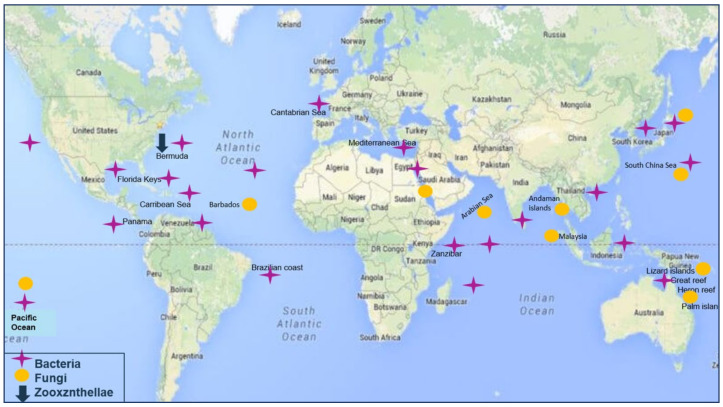
Geographical distribution of isolated hard corals-associated organisms.

The associated micro-organisms showed diverse geographical distribution. Associated bacteria and fungi are mainly collected from regions of East Africa up to the Southern East of Asia, as well as the East of Australia with bacterial strains being more abundant in the North Atlantic Ocean at the North of Southern America (Figure 2).

**Figure 3:**
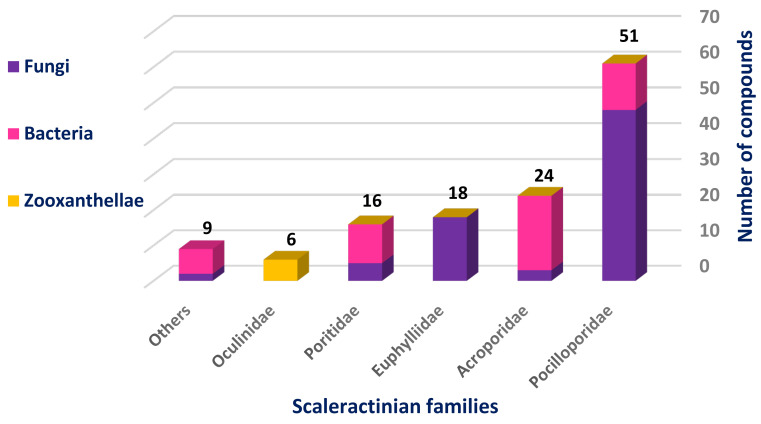
Number of compounds isolated from different studied Scleractinian families-associated organisms.

The most studied Scleractinian family was Pocilloporidae with 51 isolated compounds, 80% of which were isolated from different fungal strains, while the least studied one was Oculinidae, which was confined only to Zooxanthellates and gave rise to six compounds (Figure 3).

**Figure 4:**
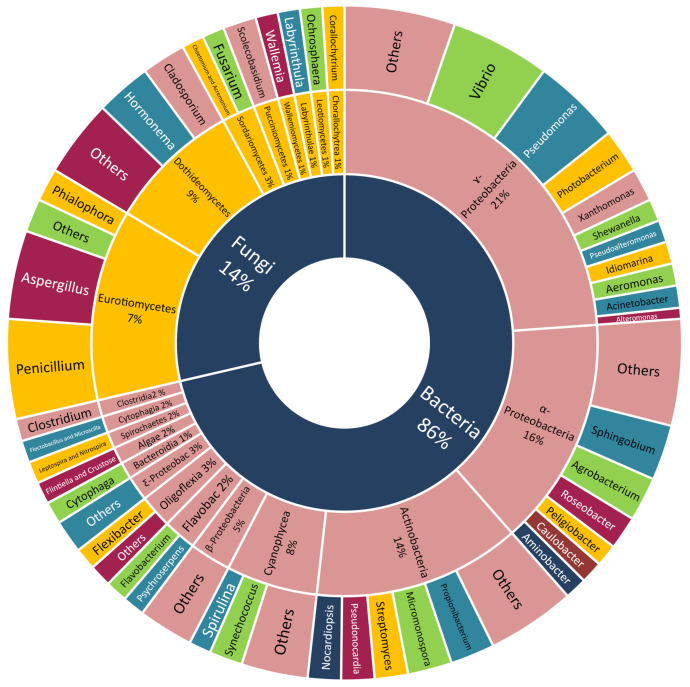
Scleractinia-associated organisms studied genera without isolated compounds.

Many classes of Scleractinian-associated microorganisms were cultivated and identified but with no further chemical investigation. The bacteria attained the largest portion (86%), with the class γ-Proteobacteria being the most easily culturable one (21%), α-Proteobacteria (16%), Actinobacteria (14%), Cyanophyceae (8%), and the others being equally distributed between the respective genera. The Scleractinian-associated fungi were less easily cultivated (14%), with the two most reported classes, Dothideomycetes (9%) and Eurotiomycetes (7%) (Figure 4).

Although the largest portion of Scleractinian-associated microorganisms were only identified, a vast number was also subjected to chemical investigation and led to the isolation and structural elucidation of 134 compounds up to date. From different fungal strains, 84 compounds (63% of the total number) were obtained, 61 of them were received from those belonging to Sordariomycetes, with most of them (46 compounds) isolated from the genus *Scopulariopsis*, the other 15 compounds were obtained from the genus *Gliomastix*. The remaining 23 compounds were found in *Aspergillus* (18 compounds) and *Talaromyces* (five compounds), both belonging to the Eurotiomycetes.

**Figure 5:**
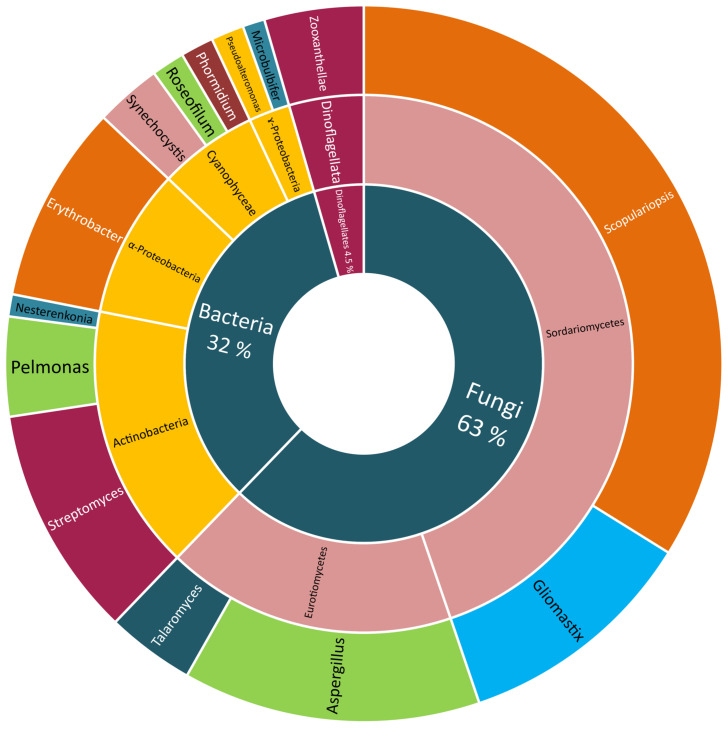
Scleractinia-associated organisms studied genera with isolated compounds.

On the other hand, more bacterial strains were studied though producing a smaller number of metabolites (44 compounds). From Actinobacteria, 21 compounds were isolated, with 14 metabolites from *Streptomyces*, six from *Pelmonas* and only one from *Nesterenkonia*. This was followed by the class α-Proteobacteria, from which twelve compounds were obtained from *Erythrobacter*, and the class Cyanophyceae, from which eight metabolites were obtained (four from *Synechocystis* sp., and the other four compounds isolated from both *Roseofilum* sp. and *Phormidium* sp.). The least studied bacterial class was the γ-Proteobacteria with only three isolated compounds from the two studied species *Pseudoalteromonas* (two constituents) and *Microalbu* (one compound). The zooxanthellae were the least studied Scleractinian-associated microorganisms with only six isolated compounds (5%) (Figure 5).

**Figure 6:**
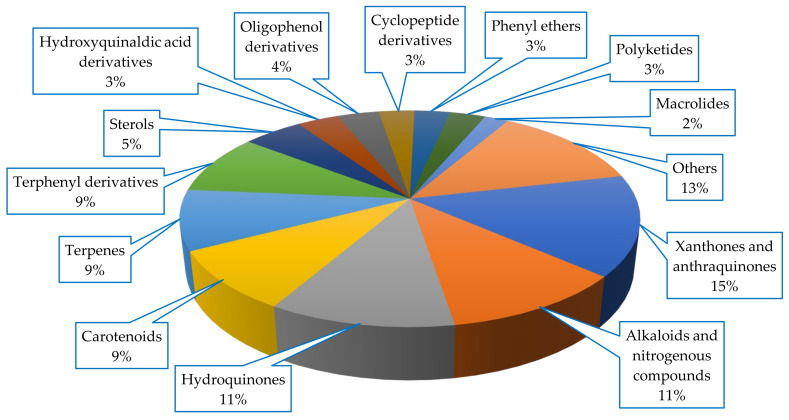
Chemical skeletons of compounds isolated from Scleractinia-associated organisms.

Diverse classes of compounds were isolated from Scleractinia-associated microorganisms, with xanthones and anthraquinones being the most abundant metabolites (15%), all of which were isolated from fungal strains (Figure 6).

**Figure 7:**
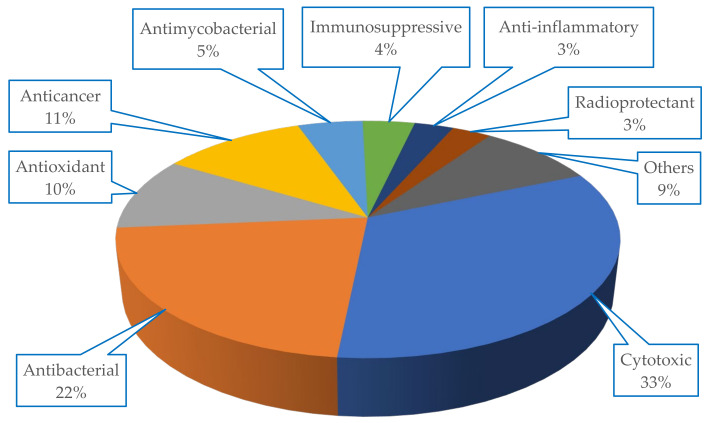
Biological activity spectrum of compounds isolated from Scleractinia-associated organisms.

The metabolites isolated from Scleractinia-associated organisms exhibited diverse biological activities, with more than 50% of the isolated compounds having cytotoxic properties (33%), followed by antibacterial (22%) potencies (Figure 7).

## 2. Scleractinia-Associated Fungi

**Figure 8:**
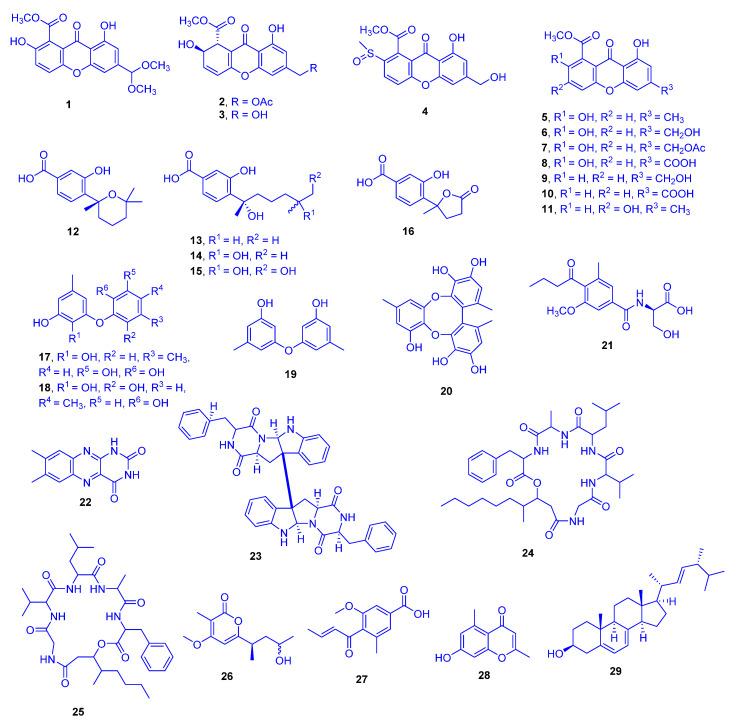
12-Dimethoxypinselin (**1**), 12-*O*-acetyl-AGI-B4 (**2**), AGI-B4 (**3**), hyperxanthone C (**4**), pinselin (**5**), sydowinin B (**6**), 13-*O*-acetyl-sydowinin B (**7**), compound **8**, sydowinin A (**9**), compound **10**, compound **11**, sydowic acid (**12**), sydonic acid (**13**), 11-hydroxysydonic acid (**14**), 11,12-dihydroxysydonic acid (**15**), 1-hydroxyboivinianic acid (**16**), violaceol I (**17**), violaceol II (**18**), diorcinol (**19**), rikuzenol (**20**), scopulamide (**21**), lumichrome (**22**), WIN 64821 (**23**), scopularide A (**24**), scopularide B (**25**), scopupyrone (**26**), pyrenochaetic acid (**27**), 7-OH-2,5-dimethylchromone (**28**) and ergosterol (**29**).

In the aqueous methanol extract of solid rice culture of *Scopulariopsis* sp., which had been isolated from the inner tissue of the host coral *Stylophora* collected from the Red Sea in Egypt, 29 different compounds were detected. They comprised of eleven xanthones (compounds **1**–**11**), five sesquiterpene derivatives **12**–**16**, four phenyl ethers **17**–**20**, five alkaloids **21**–**25**, and four miscellaneous compounds **26**–**29** (Figure 8). Compounds **1**, **2**, **15**, **16**, **21,** and **26** were new, while **6** and **14** had previously been isolated from the host coral *Stylopora* itself. Remarkably, the isolation of compounds **6** and **14** from the *Stylophora* extract evidences that production of congeneric compounds may go on when the fungus is restricted within its host [23]. The ethyl acetate extract of this fungus cultivated on solid rice medium was found to display cytotoxicity against the mouse lymphoma cell line L5178Y [23]. Cytotoxicity investigations of the isolated compounds against the mouse lymphoma cell line (L5178Y) highlighted compounds **3**, **17**, **18**, and **24** as significant with IC_50_ values of 1.5, 9.5, 9.2, and 1.2 µM, respectively, compared to that of kahalalide F with an IC_50_ value of 4.3 µM as a reference drug. The remaining compounds were not cytotoxic to mouse lymphoma L5178Y cells.

Structure activity relationship (SAR) studies showed that the cytotoxicity of AGI-B4 (**3**) and the lack of activity for the remaining xanthone derivatives **1**–**2** and **4**–**11**, indicated that both the dihydroxanthenone nucleus and a C-12 free hydroxy group are operational landscapes that are imperative for the cytotoxic activity. Comparison between scopularide A (**24**) and scopularide B (**25**) suggested that the length of the aliphatic side chain and hence possibly the lipophilicity are also important for the activity. Concerning the cytotoxicity of the biphenyl ether derivatives violaceol I (**17**) and violaceol II (**18**) and the absence of such activity for diorcinol (**19**), led to the hypothesis that increasing the number of hydroxy groups in these molecules boosted the cytotoxic activity [24].

In an earlier study, pinselin (**5**) exhibited significant immunosuppressive activity against Con-A induced (T cell) and LPS-induced (B cell) proliferations of mouse splenic lymphocytes, with IC_50_ values of 5.1 and 7.4 µg/mL, respectively (azathioprine was the reference drug with IC_50_ value of 2.7 µg/mL in both types). A comparable study investigated the immunosuppressive activity of sydowinin A (**9**) and sydowinin B (**6**) revealed the strong potency of sydowinin A (**9**), with IC_50_ values of 6.5 and 7.1 µg/mL, respectively, while sydowinin B (**6**) was moderately active with IC_50_ values of 19.2 and 20.8 µg/mL, respectively [25]. SAR studies suggested that the presence of the free OH group at position 2 in pinselin (**5**) [26] might be important for the appearance of the activity. It was already known that the suppressive effects of substituted xanthones against the proliferation of human lymphocytes were ascribable to the positions of substituents on the xanthone nucleus [26].

**Figure 9:**
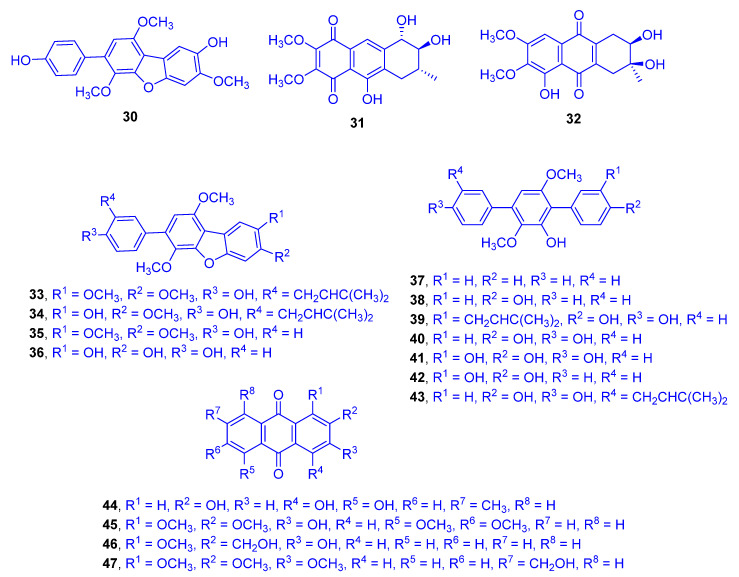
4-Methylcandidusin A (**30**), aspetritone A (**31**), aspetritone B (**32**), 3,4-dimethyl-3″-prenylcandidusin A (**33**), 4-methyl-3″-prenylcandidusin A (**34**), 3,4-dimethylcandidusin A (**35**), candidusin A (**36**), 4,4′-deoxyterphenyllin (**37**), 4″-deoxyterphenyllin (**38**), 3-prenylterphenyllin (**39**), terphenyllin (**40**), 3-hydroxyterphenyllin (**41**), 3-hydroxy-3″-deoxyterphenyllin (**42**), 3″-prenyl-terphenyllin (**43**), emodin (**44**) and compounds **45**–**47**.

4-Methylcandidusin A (**30**), aspetritone A (**31**), and aspetritone B (**32**), as well as 15 known compounds including prenylcandidusin derivatives, terphenyllin analogues, and anthraquinone congeners (compounds **33**–**47**) were detected from the ethyl acetate extract of the culture of the coral-derived fungus *Aspergillus tritici* SP2-8-1 (Figure 9). The fungus was isolated from the Scleractinian coral *Galaxea fascicularis* (family: Euphylliidae) collected at Port Dickson, Malaysia, and identified by ITS sequence homology [27]. Aspetritone A (**31**), aspetritone B (**32**), and 3-prenylterphenyllin (**39**) showed significant cytotoxicity against HeLa, A549, and HepG2 cell lines. Compound **31** attained IC_50_ values of 2.67, 3.13, and 3.87 µM, respectively. Compound **32** reached IC_50_ values of 10.57, 4.67, and 8.57 µM, while compound **39** achieved IC_50_ values of 3.23, 3.87, and 2.1 µM, respectively. The cytotoxic activities of compounds **31**, **32**, and **39** were compared with those of doxorubicin as a reference drug (IC_50_ values of 0.5, 0.09, and 1.06 µM, respectively). The other compounds showed moderate cytotoxicity, with IC_50_ values ranging from 13 to 19.7 µM except for compounds 3,4-dimethylcandidusin A (**35**), 4,4′-deoxyterphenyllin (**37**), 3-hydroxy-3″-deoxyterphenyllin (**42**), 3-hydroxy-2-hydroxymethyl-1-methoxyanthracene-9,10-dione (**46**) and 1,2,3-trimethoxy-7-hydroxy- methylanthracene-9,10-dione (**47**), which were totally inactive. Compound **31** and 4-methyl-3″-prenylcandidusin A (**34**) were significantly active against two strains of methicillin-resistant *S. aureus* (MRSA) *(*including ATCC 43300 and CGMCC 1.12409 strains), with MIC values of 7.53 and 7.63 µg/mL, 3.8 and 3.8 µg/mL, respectively, compared to chloramphenicol as a reference drug, with MICs of 7.67 and 7.87 µg/mL, respectively. Compound **33** was strongly active against the methicillin-resistant CGMCC 1.12409 strain, but moderately active against MRSA ATCC 43300 strain with MICs of 7.57 and 15.67 µg/mL, respectively. Compounds **32** and **39** as well as emodin (**44**) were moderately active against both strains of MRSA, with MIC values ranging around 15 µg/mL. Compound **30,** candidusin A (**36**), 4″-deoxyterphenyllin (**38**), compound **40**, 3-hydroxyterphenyllin (**41**), 3″-prenylterphenyllin (**43**), and 3-hydroxy-1,2,5,6-tetramethoxyanthracene-9,10-dione (**45**) were inadequately active while other compounds totally lacked such an activity.

SAR studies indicated that prenylation of terphenyllin or candidusin and the cyclohexene moiety in anthraquinone derivatives may influence their bioactivity. *C*-prenylation, which plays an important role in diversification of natural bio compounds in polyhydroxy-p-terphenyl analogues, as 3,4-dimethyl-3″-prenylcandidusin A (**33**), 4-methyl-3″-prenylcandidusin A (**34**), 3-prenylterphenyllin (**39**) and 3″-prenylterphenyllin (**43**), critically influences their cytotoxicity and antibacterial activities, compared to the un-prenylated terphenyllin and candidusin derivatives. Additionally, the special cyclohexene moiety positioned within compounds **31**, **32**, and **44**–**47** also significantly affected their relatively strong bioactivity [28].

**Figure 10:**
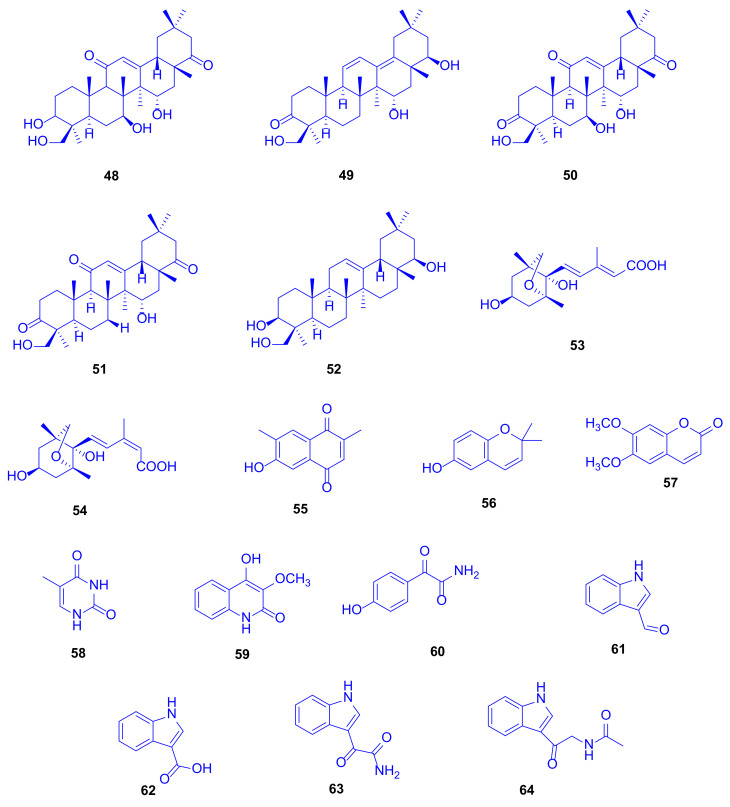
3β,7β,15α,24-Tetrahydroxyolean-12-ene-11,22-dione (**48**), 15α,22β,24-trihydroxyolean-11,13-diene-3-one (**49**), 7β,15α,24-trihydroxyolean-12-ene-3,11,22-trione (**50**), 15α,24-dihydroxyolean-12-ene-3,11,22-trione (**51**), soyasapogenol B (**52**), (2*E*, 4*E*)-4′-dihydrophaseic acid (**53**), (2*Z*, 4*E*)-4′-dihydrophaseic acid (**54**), 6-hydroxy-2,7-dimethyl-1,4-naphthoquinone (**55**), 6-hydroxy-2,2-dimethyl-2*H*-chromene (**56**), scoparone (**57**), 5-methyluracil (**58**) and compounds **59**–**64**.

A complementary study on the fungus *Scopulariopsis* sp., isolated from the hard coral *Stylophora* sp. collected near the Egyptian coastline of Ain El-Sokhna in the Red Sea, was constructed to explore its metabolic potential when grown on white beans. This growth condition was chosen due to the fact that switching the culture media from rice to white beans had already in the past caused significant changes of fungal metabolites [29]. This approach was rewarding because the metabolites isolated in that study had not been detected when the fungus was cultivated on rice medium, which strongly supports the one strain many compounds (OSMAC) approach [30]. Two new terpenoids, 3*β*,7*β*,15*α*,24-tetrahydroxyolean-12-ene-11,22-dione (**48**) and 15*α*,22*β*,24-trihydroxyolean-11,13-diene-3-one (**49**) as well as 14 known compounds, **50**–**64** (Figure 10), were isolated, including triterpenoids, coumarins, sesquiterpenoids, and polyketides. When all compounds were investigated for cytotoxicity against mouse lymphoma cells L5178Y, and for antibacterial and antitubercular activities, none of them displayed significant activity even up to a dose of 10 µg/mL [31].

Soyasapogenol B (**52**), which had originally been isolated from soybean, showed hepatoprotective effects in vitro against aflatoxin B-induced HepG2 cells at a dose of 10 µg/mL, where the considered SAR studies revealed that both the OH group and the oxygen atom at C-3 and C-24 are crucial for the hepatoprotective potential [32]. Soyasapogenol B (**52**) has also been documented to attain antimutagenic, antiviral, and anti-inflammatory activities [33]. 6-Hydroxy-2,2-dimethyl-2*H*-chromene (**56**) can be prepared via many pathways including microwave-assisted synthesis and oxidative cyclization. It proved to exhibit antioxidant and cancer protecting effects [34].

**Figure 11:**
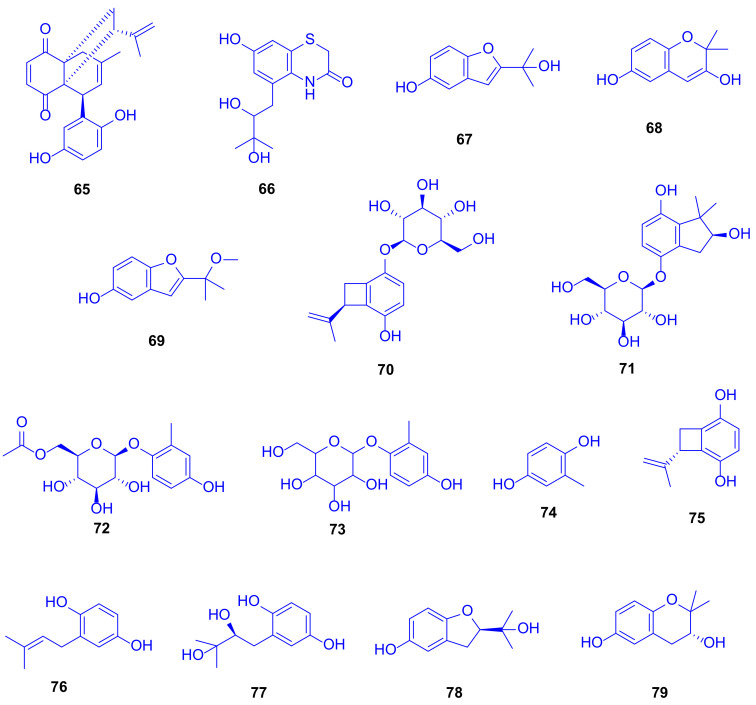
Gliomastin A (**65**), gliomastin B (**66**), gliomastin C (**67**), gliomastin D (**68**), 9-*O*-methylgliomastin C (**69**), acremonin A 1-*O*-β-D-glucopyranoside (**70**), gliomastin E 1-*O*- β-D-glucopyranoside (**71**), 6′-*O*-acetyl-isohomoarbutin (**72**), isohomoarbutin (**73**), 2-methyl-1,4-benzenediol (**74**), acremonin A (**75**), prenylhydroquinone (**76**), F-11334A_1_ (**77**), compound **78** and compound **79**.

Eight new hydroquinone derivatives, gliomastins A–D (**65**–**68**), 9-*O*-methylgliomastin C (**69**), acremonin A 1-*O*-*β*-D-glucopyranoside (**70)**, gliomastin E 1-*O*-*β*-D-glucopyranoside (**71**), and 6′-*O*-acetyl-isohomoarbutin (**72**), together with seven identified analogues (**73**–**79**) were spotted in the crude extract of the solid rice culture of the marine-derived fungus *Gliomastix* sp., which was isolated from the scleractinian coral *Stylophora* sp. (family: Pocilloporidae), collected from the Red Sea in Egypt (Figure 11) [35]. This extract displayed cytotoxic potential against the L5178Y mouse lymphoma cell line with an inhibition of 69.1% at a dose of 10 µg/mL.

Performing TDDFT-ECD (Time-dependent density functional theory-electrostatic discharge) and OR (odds ratio) calculations to determine the absolute configurations of the new compounds emphasized the novelty of compound **65** as biogenetically derived from a Diels–Alder reaction between derivatives of compounds acremonin A (**75**) and F-11334A_1_ (**77**), and gliomastin B (**66**) as a sporadically occurring sulfur-containing alkaloid derived from the identified hydroquinone, sydonic acid (**13**). The compounds gliomastin A (**65**), 2-methyl-1,4-benzenediol (**74**), prenylhydroquinone (**76**), and F-11334A_1_ (**77**) exhibited potent cytotoxicity against the L5178Y mouse lymphoma cell line, with IC_50_ values of 1.8, 1, 1.1, and 3 µM, respectively, while compound **75** was moderately active with IC_50_ value of 9.6 µM, compared to kahalaide F as a reference drug, with a IC_50_ value of 4.3 µM (Figure 11).

Compounds **74** and **75** exerted strong cytotoxicity while their glycosides **72**, **73**, and **70** were completely inactive. The ether bridge in **78** and **79** led to an entire loss of cytotoxicity compared to the parent compound **77**. When tested for antitubercular activity, compounds **67**, **74**, **76**, and **77** showed moderate activity with MIC value of 12.5 µM, while compound **75** was weekly active with a MIC value of 25 µM (rifampin was used as a reference drug, with a MIC value of <0.64 µM). SAR examination showed the importance of both, the furan ring and the free hydroxy group in compound **67** (which lacked cytotoxicity) compared to compounds **68**, **78**, and **79**, which lacked anti-tubercular activity. Testing the antibacterial potential revealed that compounds **74** and **76** were significantly active, with MICs ranging from 6.25 to 10.12 µM, compared to moxifloxacin as a reference drug, with a MIC value of 1.56 µM [35]. Remarkably, although compound **73** lacked any reported biological activity, it has long been used in traditional Chinese medicine for treatment of heart diseases (Figure 11) [36].

**Figure 12:**
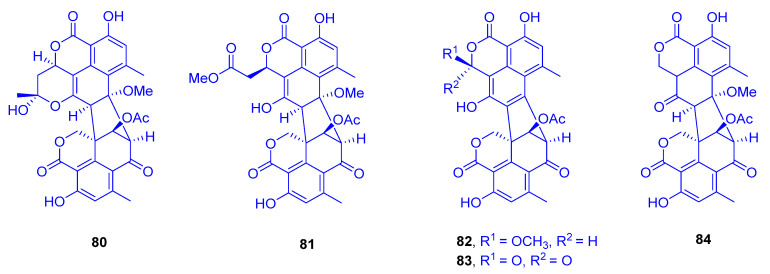
Verruculosin A (**80**), verruculosin B (**81**), bacillisporin F (**82**), duclauxin (**83**) and xenoclauxin (**84**).

Two oligophenalenone dimers, verruculosin A (**80**) and verruculosin B (**81**), along with three known analogues, bacillisporin F (**82**), duclauxin (**83**), and xenoclauxin (**84**), all of which possess a unique octacyclic skeleton, were obtained from the marine-derived fungus *Talaromyces verruculosus*, which was isolated from *Goniopora* sp. (family: Poritidae) (Figure 12) [37]. Interestingly, despite the structural resemblance between all the heptacyclic polyketides **80**–**84**, they displayed remarkable variances in their activity spectra. In the research for novel anti-tumor agents, compounds **80**, **82**, **83**, and **84** showed potent CDC25B (a specific tyrosine phosphatase) inhibitory activities, with IC_50_ values of 0.38, 0.4, 0.75 and 0.26 µM, respectively; as a reference compound, Na_3_VO_4_ was used with an IC_50_ value of 0.52 µM. The outcomes designated oligophenalenone dimers as a tool for screening of the new natural CDC25B inhibitor candidates.

Exploring the anti-EGFR (epidermal growth factor receptor) activity as a new target for the same purpose, all compounds showed moderate potential, except for compound **84**, which exhibited the strongest activity, with an IC_50_ value of 0.24 µg/mL, compared to afatinib as a reference drug of 0.0005 µg/mL [37]. Remarkably, both compound **83** and its analogue **84** showed high potency against murine leukemia L-120 cells and exhibited strong inhibition on mitochondrial respiration [38,39].

Following the biosynthetic pathway of duclauxin (**83**) from *Talaromyces stipitatus*, a cascade of redox transformations was characterized to start with the dioxygenase DuxM, to proceed via redox enzymes, and to end with oxidative coupling catalyzed by a P_450_ monooxygenase [40].

## 3. Scleractinia-Associated Bacteria

**Figure 13:**
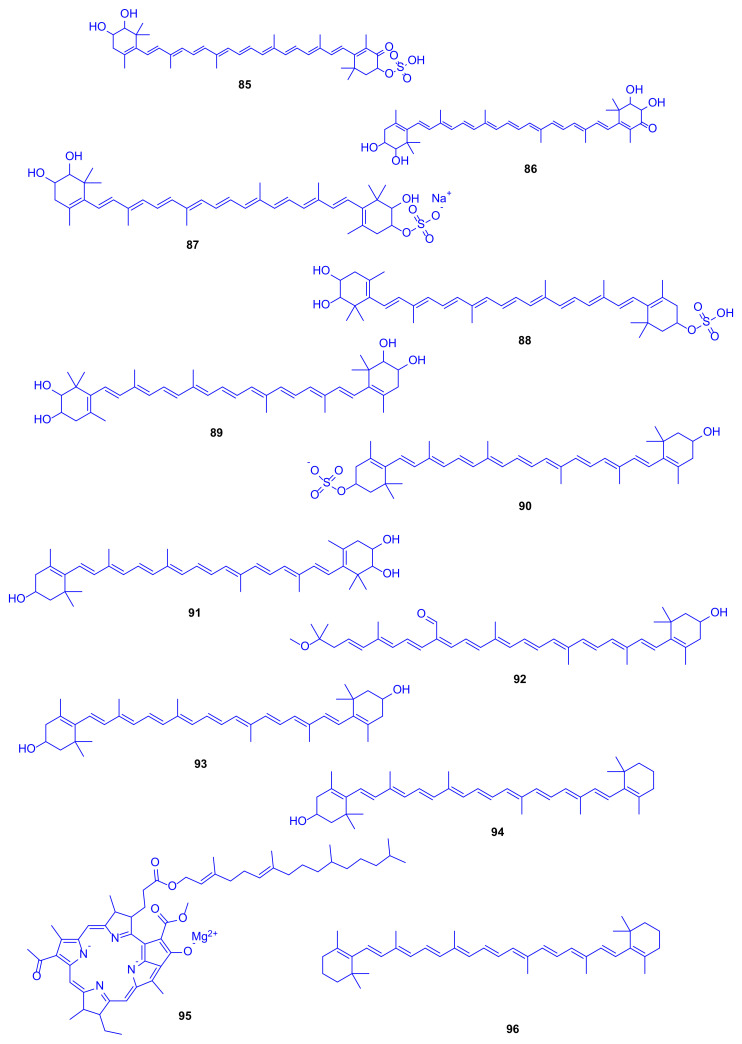
Erythroxanthin sulfate (**85**), ketonostoxanthin (**86**), nostoxanthin sulfate (**87**), caloxanthin sulfate (**88**), nostoxanthin (**89**), zeaxanthin sulfate (**90**), caloxanthin (**91**), bacteriorubixanthinal (**92**), zeaxanthin (**93**), β-cryptoxanthin (**94**), bacteriochlorophyll (**95**) and β-carotene (**96**).

*Erythrobacter flavus* strain KJ5 (formerly called *Erythrobacter* sp. strain KJ5) is a yellowish aerobic marine bacterium, which was isolated from the hard coral *Acropora nasuta* (family: Acroporidae) in the Karimunjawa Islands, Central Java Province, Indonesia. It is worth to mention that previous phylogenetic analysis utilizing 16S rDNA sequence had shown that this species has a genetic similarity of 96% to *E. flavus* [41]. The genus *Erythrobacter* was first classified by Shiba and Simidu [42], and found to compromise aerobic marine photosynthetic bacteria that have bacteriochlorophyll, carotenoids as well as non-sulphated carotenoids. Analyses using HPLC, UV–VIS, and FTIR spectrophotometers, and MS/MS with electrospray ionization were employed to identify carotenoids isolated from this strain including compounds **85**–**96** (Figure 13), with caloxanthin sulfate (**88**) and nostoxanthin (**89**) as the most abundant representatives. An enzyme activity assay was performed to investigate the unique existence of such compounds, leading to the discovery of sulfotransferases that catalyze the conversion of carotenoids into carotenoid sulfates using the cell-free extract as well as incubation, which may be responsible for the antithrombotic, antifouling, antiviral, and anti-inflammatory activities [43,44,45]. Although hydrogen sulfide is toxic to a wide range of eukaryotic organisms and has also led to the commencement of the coral black band disease, some studies have suggested that the co-existence of sulfur-oxidizing bacteria might convert sulfide to sulfate, which leads to coral health benefits, including corals belonging to the genus *Acropora* [46].

**Figure 14:**
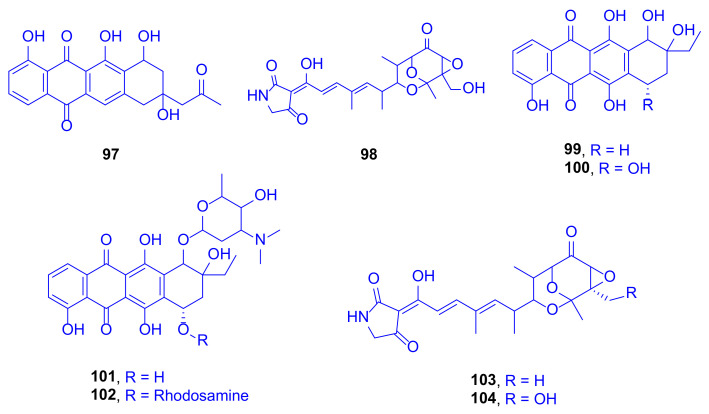
Aranciamycin K (**97**), isotirandamycin B (**98**), γ-rhodomycinone (**99**), β-rhodomycinone (**100**), 262-6 (**101**), β-rhodomycin-II (**102**), tirandamycin A (**103**) and tirandamycin B (**104**).

A bioassay-guided fractionation study of the culture extracts of *Streptomyces* sp. SCSIO 41399, cultivated from the Scleractinian coral, *Porites* sp. collected from the Wenchang (Hainan Province, China), led to isolation and identification of eight compounds using spectroscopic data and ECD calculations. These compounds included one new anthracycline, aranciamycin K (**97**), one new tirandamycin analogue, isotirandamycin B (**98**), as well as known four anthracycline derivatives, γ-rhodomycinone (**99**), β-rhodomycinone (**100**), compound 262-6 (**101**), and β-rhodomycin-II (**102**), besides the two known tirandamycin derivatives tirandamycin A (**103**) and tirandamycin B (**104**) (Figure 14). In view of the necessity to treasure new antibiotics, and due to the finding that tirandamycins had demonstrated activity against vancomycin-resistant *Enterococcus faecalis*, the compounds were tested against *Streptococcus agalactiae*. Compounds **98**, **103**, and **104** were highlighted as potent bacteriostatic leads, with MIC values of 5, 2.5, and 2.5 μg/mL, respectively (erythromycin is a reference drug with an IC_50_ value of 5 μg/mL) [47].

Investigation of compounds **97**–**104** for their in vitro cytotoxicity against the K562 cell lines revealed that compound **101** showed high potency, with an IC_50_ value of 1.8 μM, while compounds **99** and **102** exhibited moderate cytotoxic activities, with IC_50_ values of 22 and 12.1 μM, respectively (Paxlitaxel is a reference drug with an IC_50_ of 0.21 μM). However, when these compounds were tested against five pathogenic fungi, *Colletotrichum gloeosporioides*, *C. asianum*, *C. acutatum*, *Fusarium oxysporum*, and *P. oryza*, no activity was spotted [48].

Remarkably, the first example of bacterial biosynthetic P_450_ enzymes (TAM I and TAM L) with a versatile activity was reported after revealing that they catalyze a multi-step oxidative cascade of tailoring tirandamycin antibiotics. The cascade starts with tirandamycin C, passes through tirandamycins E and D, followed by successive hydroxylation and epoxidation, to give tirandamycin A (**103**) and tirandamycin B (**104**) [49]. Such follow-up modifications in the tirandamycin pathway contribute pointedly to the antibiotic potency, where the SAR found that the MIC of tirandamycin C was 110 µM, far weaker than that of tirandamycin A (MIC = 2.25 µM) [47]. This makes the previously mentioned tailoring steps biologically significant, and tunes towards the production of tirandamycin A, the most potent among all the tirandamycin antibiotics [49].

**Figure 15:**
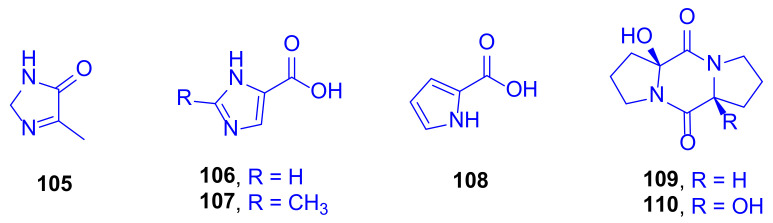
Pelopuradazole (**105**), 3*H*-imidazole-4-carboxylic acid (**106**), 2-methyl-3*H*-imidazole-4-carboxylic acid (**107**), 1*H*-pyrrole-2-carboxylic acid (**108**), pelopurin A (**109**) and pelopurin B (**110**).

The new imidazole alkaloid pelopuradazole (**105**), the three known imidazole alkaloids 3*H*-imidazole-4-carboxylic acid **106**, 2-methyl-3*H*-imidazole-4-carboxylic acid **107**, and 1*H*-pyrrole-2-carboxylic acid **108**, as well as the two known cyclo-dipeptides pelopurin A (**109**) and pelopurin B (**110**) were isolated from a culture of *Pelmonas puraquae* sp. associated with the coral *Acropora* sp. (family: Acroporidae) (Figure 15) [50].

Compound **108** showed strong antibacterial inhibition against *Ralstonia solanacearun* with a MIC value of 2 µg/mL [51]. Markedly, this is the first time to isolate compounds **109** and **110** as natural products after they had previously been reported as merely synthetic compounds prepared from amino acids [52].

**Figure 16:**
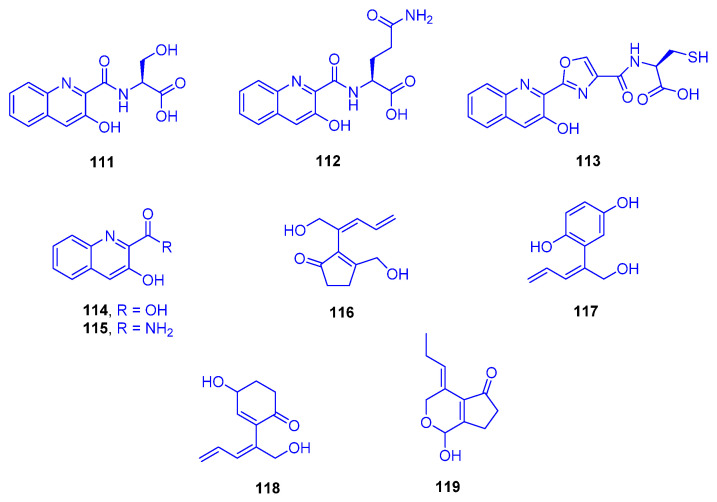
Compounds **111**–**113**, 3-hydroxyquinaldic acid (**114**), 3-hydroxyquinaldic acid amide (**115**), nakienone A (**116**), nakitriol (**117**), nakienone B (**118**) and nakienone C (**119**).

A chemical investigation was conducted on a culture of the marine derived bacterium *Streptomyces cyaneofuscatus* M-157, isolated from a solitary coral belonging to the order Scleractinia, collected at 2000 m depth in the Aviles Canyon, Cantabrian Sea (Biscay Bay, Northeast Atlantic). From the fermentation culture of that strain, five hydroxyquinaldic acid derivatives were isolated, compounds **111**–**115** and their absolute stereostructures were determined by advanced Marfey’s analysis followed by HPLC showing that the α-amino acid building blocks were L-serine, L-glutamine, and L-cysteine (Figure 16). Cytotoxic evaluation of these compounds against the human tumor cell line HepG2 highlighted 3-hydroxyquinaldic acid amide (**115**) and compound **111** as having moderate and weak activities with IC_50_ values of 15.6 and 51.5 µM, respectively, while the other compounds did not show activity even up to a concentration of 20 µg/mL [53].

Biosynthetically, compound **113** is most probably comes from compound **111**, by elongation of the peptide sequence with an L-cysteine residue and post-translational hetero-cyclization of the serine portion onto the carbonyl group of quinaldic acid to form an oxazole ring. Moreover, compounds **111**–**113** were found to possess structural features that are compatible with being biosynthetic precursors of larger chromodepsipeptides, similar to known antibacterial or cytotoxic agents, such as the luzopeptins, quinaldomycin, sandramycin, or thiochoraline, whose presence in traces in the extract would explain its antimicrobial properties [53].

From time to time, marine algal outgrowths indicate a destructive contributing factor leading to disintegration of hard coral communities. Large and concentric zones of *Synechocystis* sp. overgrowth were discovered at a depth of 1 to 2 m in the waters off Yonaine at Nakijin Village (Okinawa), on dark, dead, and necrotic branches of *Acropora* sp. (family: Acroporidae). Four new cyclic C_11_ compounds were isolated from this microalgal-coral assemblage nakienone A (**116**), nakitriol (**117**), nakienone B (**118**), and nakienone C (**119**), which are assumed to be responsible for the chemical ecology of such a necrotic interaction (Figure 16). Nakienone A (**116**) was found to be cytotoxic to both KB and HCT 116 cell lines, with LD_50_ values of 5 and 20 µg/mL, respectively, while nakitriol (**117**) displayed non-selective cytotoxicity to DNA repair defective cell lines EM9, XRS-6, UV20, and BRI at LD_50_ of 20 µg/mL [54].

**Figure 17:**
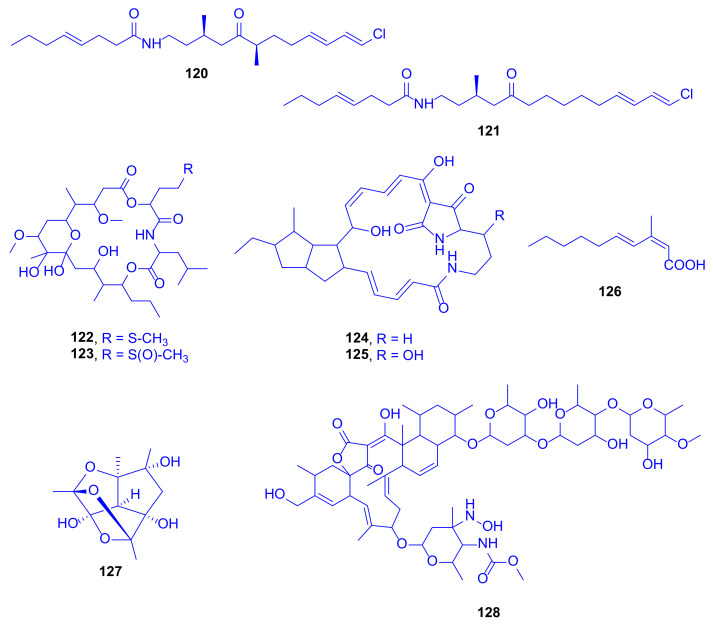
Pitiamide A (**120**), 1*E*-pitiamide B (**121**), looekeyolide A (**122**), looekeyolide B (**123**), alteramide A (**124**), alteramide B (**125**), (2*Z*,4*E*)-3-methyl-2,4-decadienoic acid (**126**), nesteretal A (**127**) and lobophorin K (**128**).

In the same context, the new fatty acid amides pitiamide A (**120**) and 1*E*-pitiamide B (**121**) were purified following a bioassay-guided fractionation from *Phormidium corallyticum* (a mixed cyanobacterial infection) recovered from *Porites cylindrica* (family: Poritidae). The cyanobacterium was collected from the shallow reef flat at Piti Bomb Holes (Guam, Mariana Islands) (Figure 17). The black band disease infection was detected in Micronesia on the tops of the reef-building coral in the form of a thin blue-gray tuft-forming assemblage of both *Lyngbya majuscule* and *Microcoleus* sp. [55].

Both pitiamide A (**120**) and 1*E*-pitiamide B (**121**) exhibited significant antiproliferative activities towards HCT116 colorectal cancer cells with IC_50_ values of 1.1 and 5.1 µM, respectively, and by comparison with vinblastine as a reference drug (IC_50_ = 1.2 nM). Interestingly, the dose-response curves of 1*E*-pitiamide B (**121**) were consistently rightward shifted 4 to 5-fold compared with that of pitiamide A (**120**), signifying a SAR between the two of them. It was found that either the absence of an α-ketone methyl or the increase in the span of the methylene chain triggered an increase of the IC_50_ to a higher value [55]. Many fatty acid amides from marine cyanobacteria exhibit various neuro-modulatory activities, possibly owing to their structural features, which may modify ion channels or receptors on cell membranes [56,57]. Consequently, pitiamide A (**120**) was further evaluated for its effects on the plasma membrane potential and on intracellular calcium in HCT116, causing plasma membrane hyperpolarization at 32 µM and 1 µM, with no effect being observed at 32 nM [58]. Petiamide A (**120**) was synthesized by applying Evans’ oxazolidinone alkylation, a novel water-accelerated modification of Negishi’s zirconocene-catalyzed carbometallation as well as Mitsunobu’s alkylation of a nosyl-activated amide [59].

Looekeyolide A (**122**), a new 20-membered macrolide, and its autoxidation product looekeyolide B (**123**) were first identified from lipophilic extracts of black band disease (BBD), a lethal, polymicrobial disease consortium dominated by the cyanobacterium *Roseofilum reptotaenium*, isolated from *Montastraea* (family: Montastraeidae), collected at Looe Key Reef (Florida Keys, USA) and known to kill many coral types worldwide (Figure 17). The biosynthesis of the two compounds is presumed to occur through the well conserved nonribosomal peptide (NRP)/polyketide (PK) hybrid biosynthetic cluster, which was detected in four *Roseofilum* metagenome-assembled genomes (MAGs) previously analyzed and is predicted to produce looekeyolide A (**122**) [60]. Looekeyolide A (**122**) is easily autoxidized to give looekeyolide B (**123**), making it challenging to assess its natural functions. Thus, compound **123** was tested directly in various assays, where it did not show any cytotoxic or antibacterial activity [61]. Looekeyolide A (**122**) may play a role in reducing H_2_O_2_ and other reactive oxygen species (ROS) that could occur in the BBD layer as it overgrows and causes bleaching of both inner and outer layers of coral tissue as well as destruction as previously occurred in the scleractinian coral *Stylophora pistillata* (family: Pocilloporidae) [62]. Over 20 μmol/L H_2_O_2_ has been detected in the immediate coral diffusive boundary layer, which may aid corals in removing some of the internal H_2_O_2_ produced by their endosymbiotic algae and possibly have a self-protective function. Looekeyolide A (**122**) may allow *Roseofilum* to cope with this coral-produced source of H_2_O_2_ as it migrates across the coral colony, and reduces the H_2_O_2_ concentration to nearly zero when being incubated with a 1 mM looekeyolide mixture [61].

The first attempt for isolation and identification of antagonistic bacteria resulted in the isolation of *Pseudoalteromonas* sp. strain S1 from *Montipora* digitate collected from shallow water reefs (<1 m) around the Tropical Biosphere Research Center Sesoko, Station of University of the Ryukyus in the Okinawa Islands (Japan). Two tetracyclic alkaloids, alteramides A (**124**) and B (**125**) were obtained and identified, although alteramide A (**124**) was originally obtained from the chloroform/methanol extract of the bacterium *Alteromonas* sp. associated with the marine sponge *Halichondria okadai* collected off Nagai (Kanagawa, Japan), and grown statically in PYG broth, which suggested that the *Montipora* coral might not be the actual producer (Figure 17) [63]. Alteramide A (**124**) exhibited cytotoxicity against murine leukemia P388 cells, murine lymphoma L1210 cells, and against human epidermoid carcinoma KB cells in vitro, with IC_50_ values of 0.1, 1.7, and 5 µg/mL, respectively [64]. Both of the compounds, **124** and **125**, showed  similar  antifungal  activity, using the paper disk method, against *Mucor hiemalis* (inhibition zones 9 and 8 mm, respectively) and *Dichotomomyces cejpii* (inhibition zones 8 and 7 mm, respectively) at 10 µg/disk, but only alteramide A (**124**) showed antifungal activity against *Colletotrichum acutatum* (inhibition zone 7 mm) [64].

(2*Z*,4*E*)-3-Methyl-2,4-decadienoic acid (**126**) is a new fatty acid, detected as a major metabolite in a butanol culture broth extract of a marine bacterium *Microbulbifer* sp. C46, isolated from the stony coral *Porites* sp. (family: Poritidae), obtained from an aquarium vendor in Nagasaki (Japan, Figure 17) [65]. When tested for antimicrobial activity, it was inactive against a wide range of bacterial strains (MIC > 100 μg/mL) but weakly active against *Saccharomyces cerevisiae* S100 (MIC = 100 μg/mL). Remarkably, it was known as a synthetic compound [66], and this was the first time to be naturally isolated, besides being a biosynthetically unique metabolite. It attains an uncommon methylation pattern in its carbon chain, derived from the *C*-methylation with L-methionine at a carbon originating from a carbonyl carbon of acetate [65].

A novel, intriguing cage-like polyhemiacetal, nesteretal A (**127**), was obtained by large scale fermentation from the coral-derived actinomycete *Nesterenkonia halobia*, isolated from *Platygyra*, (family: Merulinidae) (Figure 17). Its biosynthesis is projected to comprise a thermodynamically favorable cascade cyclization process including an intramolecular aldol reaction, which is followed by three well-organized hemiacetalization steps. Nesteretal A (**127**) was investigated, at 10–40 μM concentration, for its RXRα transcriptional activity by using the reporter gene assay. It revealed a weak potential but showed an interesting competitive effect when tested with 9-cisretinoic acid, a natural RXRα ligand, at a high-dose level. When tested against five tumor cell lines of BIU-87, ECA-109, Hela-S3, PANC-1, and BEL-7402 at a concentration of 40 μM, it was totally inactive [67].

A new member of the lobophorin family, lobophorin K (**128**), was obtained from cultures of *Streptomyces* sp. M-207, previously isolated from the sclaractinian coral *Lophelia pertusa*, (family: Caryophylliidae) collected at the Cantabrian Sea at a depth of 1800 m (Figure 17). The compound exhibited a strong cytotoxicity against a human immortalized hepatocyte cell line (THLE-2), with an IC_50_ value of 6.3 µM, and a moderate one against both a human breast adenocarcinoma cell line (MCF-7) and a human pancreatic carcinoma cell line (MiaPaca-2), with IC_50_ values of 23 and 34 µM, respectively. When tested at a maximum concentration of 42.6 µM against colon (HT-29), lung (A-549), and hepatocarcinoma (HepG2) cell lines, it was completely inactive. It displayed moderate and selective activity against the Gram-positive bacteria methicillin-sensitive *S. aureus* EPI167 MSSA, with a MIC_90_ value of 40–80 µg/mL [68].

## 4. Scleractinia-Associated Zooxanthellae

**Figure 18:**
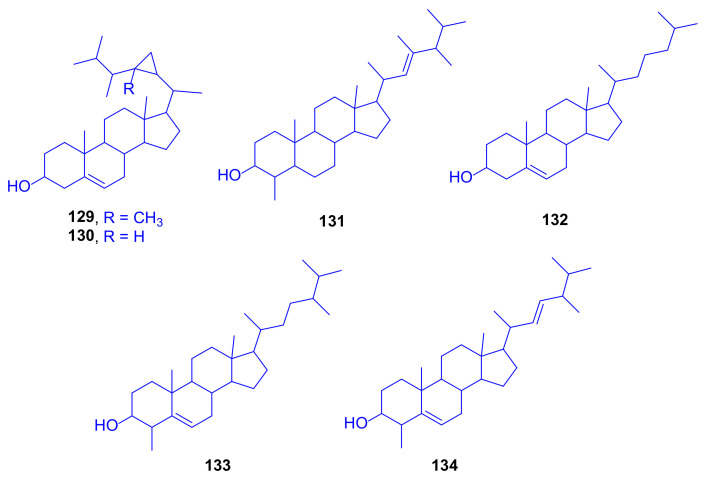
Gorgosterol (**129**), 23-desmethyl-gorgosterol (**130**), dinosterol (**131**), cholesterol (**132**), 4α-(24S)-dimethyl-cholesta-3β-ol (**133**) and 4α-(24R)-dimethyl-cholesta-22-en-3β-ol (**134**).

An early American study firstly isolated 23-methylated dinoflagellate sterols from zooxanthellae, and stated that the patterns of synthesized sterols can be used as taxonomic markers for the strains of gorgonian zooxanthellae. A number of marine sterols with a cyclopropyl side chain was recognized from cultured zooxanthellae isolated from *Oculina diffusa* (family: Oculinidae) from Permuda. The study revealed that an asymbiotic algae can synthesize the unique marine sterols, gorgosterol (**129**) (which can be derived biosynthetically from 5-dehydrodinosterol) and 23-desmethylgorgosterol (**130**), together with dinosterol (**131**), cholesterol (**132**), 4α-(24S)-dimethylcholesta-3β-ol (**133**) and 4α-(24R)-dimethylcholesta-22-en-3β-ol (**134**) (Figure 18) [69]. In an attempt to explain the biosynthetic pathway of gorgosterol, cell-free extracts of the dinoflagellates *Cryptothecodinium cohnii*, *Peridinium foliaceum*, and the cultured zooxanthella symbiont of *Cassiopea xamachana* were analyzed for sterol methyltransferases using [8*H*]-*S*-adenosylmethionine via radiochemical conversions. The results demonstrated that the attenuation of gorgosterol production in aposymbiotic zooxanthellae is linked to an increase in dimethylpropiothetin biosynthesis through a decrease in *S*-adenosylmethionine concentration [70].

## 5. Diversity of the Reported Scleractinia-Associated Microbes and Their Secondary Metabolites

Phylogenetic analysis of the strain M-157 was performed by PCR via amplification of the 16S rDNA of strain M-157. After Basic Logic Alignment Search Tool (BLAST) sequence comparison, strain M-157 showed 100% identity to *Streptomyceces cyaneofuscatus* and was sequenced being designated as *Streptomyceces cyaneofuscatus* M-157. The phylogenetic tree revealed the evolutionary relationship of strain M-157 with a group of known *Streptomyces* species [53].

Investigating bacterial strains isolated from different Scleractinian species, *Scopulariopsis* belonging to class *Sordariomycetes* is considered as the most heavily studied fungal strain which has been isolated from *Stylophora* sp. From this species, 46 compounds have been isolated, of which only 20 are biologically active. The compounds included eleven xanthones, five triterpenes, seven sesquiterpenes, four phenylether derivatives, three alkaloids, two cyclodepsipeptides, two α-pyrones, one chromone, one sterol, three polyketides and seven nitrogenous compounds, most of which exerted antibacterial, cytotoxic and immunosuppressive potencies. The fungus *Aspergillus* belonging to class Eurotiomycetes is isolated from *Galaxea* sp., from which 18 compounds were isolated including: two prenylcandidusins, three candidusins, seven terphenyllin derivatives, and six anthraquinones, from which only 13 compounds were biologically active, mostly with antibacterial and cytotoxic activities. The fungus *Gliomastix* belonging to class Sordariomycetes was isolated from *Stylophora* sp., from which 15 hydroquinone derivatives were isolated from its fungus, only six of which were bioactive exhibiting cytotoxic and antitubercular activities.

Moreover, investigating some bacterial strains isolated from different Scleractinian species, as *Erythrobacter* sp., belonging to class Alphaproteobacteria, which was isolated from *Acropora* sp., is chemically investigated to give twelve carotenoids, of which only three were active with cytotoxic and radioprotectant potencies. The class Actinomycetes was intensely investigated, where *Streptomyces* isolated from *Porites* sp. led to isolation of eight compounds (five anthracyclines and three tirandamycine), all of which showed antibacterial and cytotoxic activities. The same bacterial strain, isolated from *Lophelia* sp. and other different corals led to isolation of the cytotoxic compounds, lobophorin and 5-hydroxyquinaldic acid derivatives, two of which were cytotoxic. Moreover, the chemical investigation of *Nesterenkonia* isolated from *Platygyra* sp. led to the isolation of a polyhemiacetal with RXRα activity. The class Betaproteobacteria has rarely been investigated. As an example, *Pelmonas* sp. was isolated from *Acropora* sp. and subjected to chromatographic analysis to give four imidazole alkaloids and two cyclopeptides, most of them were inactive. The class Gammaproteobacteria has been studied relatively less intensively, here both *Pseudoalteromonas* sp. and *Microbulbifer* sp. were isolated from *Montipora* sp. and *Porites* sp., giving rise to the isolation of two cytotoxic compounds and one antibacterial fatty acid, respectively. The class Cyanophyceae was also investigated, and both *Synechocystis* sp. and *Phormidium corallyticum* were isolated from *Acropora* sp. and *Porites* sp., to give two active cytotoxic cyclic C_11_ compounds and two antiproliferative fatty acid amides, respectively. From the same class, *Roseofilum reptotaenium* was isolated from *Montastraea* and investigated to result in the identification of two inactive macrolides.

Furthermore, the zooxanthellae, isolated from *Oculina* sp., were also investigated, leading to the isolation of six sterols, which were found to be cytotoxically inactive.

In Table S1 in the Supplementary Materials, all the reported marine natural products isolated from Scleractinia-associated microbes (compounds **1**–**134**) are presented, together with their chemical categories and their biological sources (marine organisms), as well as the locations of collection of the studied marine organisms.

## 6. ADME Analysis and Molecular Docking of Scleractinia-Associated Microbial Metabolites

**Figure 19:**
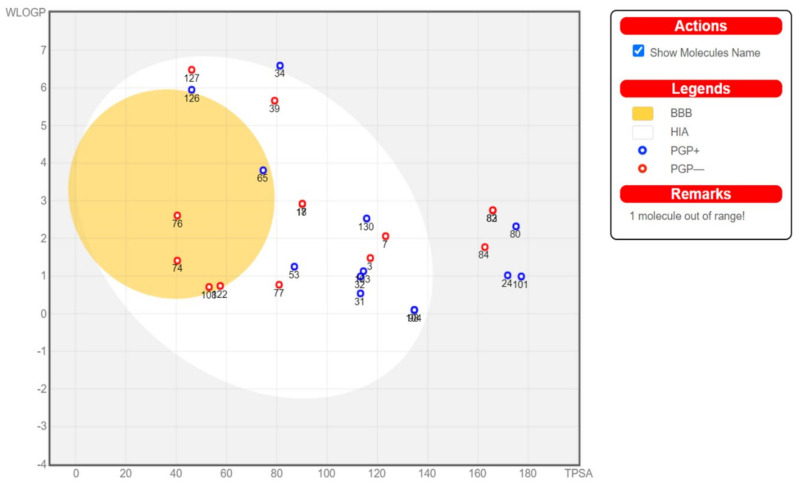
Boiled-egg diagram of the investigated compounds. BBB, passively permeate blood brain barrier; HIA, Human intestinal absorption; PGP+, compounds that effluated from central nervous system by the P-Glycoprotein; PGP-, compounds that is not binding to P-Glycoprotein.

Physicochemical properties of the investigated compounds were studies using SwissADME [71] and a boiled-egg diagram [72] was generated using the same server. Based on the ADME results, compounds were selected for further docking studies to investigate their potential effects on five SARS-COV-2 targets using Autodock Vina [73]. Compounds that showed oral absorption, which is an important criterion for a drug that is intended for mass treatment, were selected. In addition, compounds that crosses the blood brain barrier could cause several adverse effects and were excluded. Binding to P-glycoprotein (PGP+) was also studied and plotted (Figure 19) but was not used as a selection criterion to simplify the process. Investigated SARS-COV-2 targets included viral main protease (6LU7), viral methyltransferase (nsp16, 6W4H), viral RNA dependent RNA polymerase (nsp12, 7BV2), viral spike protein (6M0J), and human ACE2, which is the viral recognition protein (6VW1). Compounds and targets were prepared as reported earlier [74]. In brief, compounds were drawn or downloaded from PubChem and then were energy minimized using 1000 steps of steepest descent and conjugate gradient algorithms. Compounds were then converted to pdbqt using Autodock tools. For targets, they were retrieved from protein data bank then water molecules were removed, hydrogens and Gasteiger charges were added and non-polar hydrogens were merged. The grid box was centered on the co-crystallized ligand when available and the grid box size was adjusted to 25 × 25 × 25 Å^3^. In all cases the co-crystallized ligand was redocked to validate the docking method and its score was used as a reference to compare the tested compounds. Root mean square deviation (RMSD) between docked and crystallized ligand in the validation step was accepted when it was less than 2 Å using the DockRMSD server [75].

From the 134 compounds investigated, ADME highlighted the compounds that can be absorbed orally but without crossing the blood brain barrier (BBB) to avoid possible side effects. These target compounds are found in the egg white in the following boiled-egg diagram (Figure 19).

Based on the above criteria, 15 compounds were selected for investigating their potential effect against the above mentioned SARS-CoV-2 targets. The results of the docking are shown in Table S2 in the Supplementary Materials.

**Figure 20:**
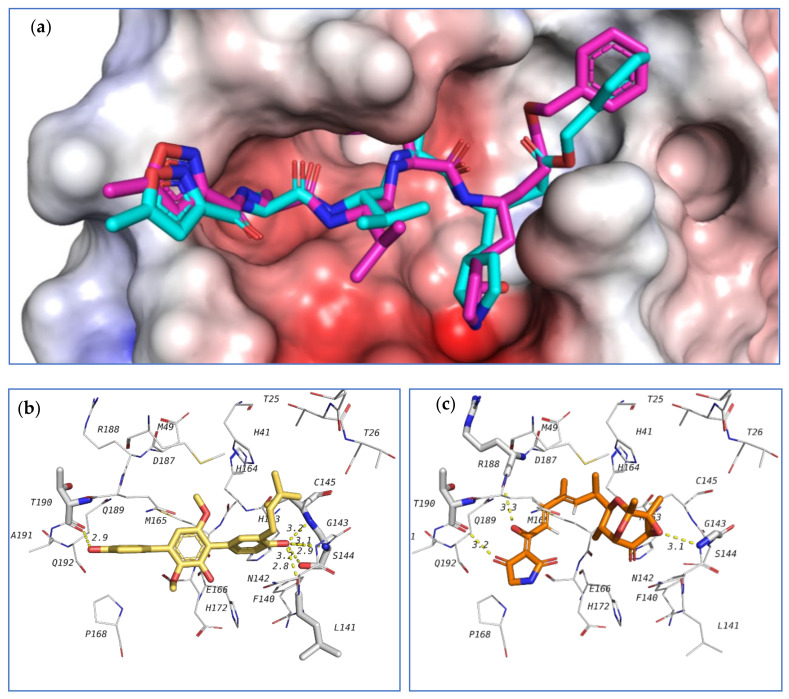
Docking results of investigated compounds in the active site of SARS-CoV-2 main protease (6LU7). (**a**) Validation of docking procedure showing good matching between crystallized (blue) and docked (pink) ligands. (**b**) Docking pose and interactions of **39** (yellow). (**c**) Docking pose and interactions of **103** (orange).

The first target was the SARS-CoV-2 main protease (M^pro^) (Figure 20). Main protease (3C-like protease) is responsible for the release of functioning peptides from the expressed polyprotein through protease activity at various locations. M^pro^ is considered as a promising target against SARS-CoV-2 due to its importance in the viral life cycle and because of the absence of human homologues [76]. The crystal structure of SARS-CoV-2 main protease (6LU7 pdb code) contains a peptide-like inhibitor called N3. The co-crystallized ligand forms hydrogen bonds with F140, G143, H163, H164, E166, D187, and T190 residues. To validate the docking method, the co-crystallized ligand was redocked in the active site and the docking algorithm was able to predict the co-crystalized ligand pose as the 3^rd^ pose, with energy score of −7.1 kcal/mol. This pose showed the least RMSD compared to the co-crystalized ligand while the pose with best docking energy followed a similar orientation with an energy score of −7.9 kcal/mol but has larger RMSD. Among the tested compounds, compounds **39** and **103** showed the same docking energy of −7.9 kcal/mol. They were both docked in the same pocket of the co-crystallized ligand N3 and formed hydrogen bonds that involved the same amino acids interacting with N3 (Figure 20). These results suggested that these two compounds, **39** and **103**, might be promising potential leads for the design of new inhibitors for viral main protease and deserve further investigation.

Another important SARS-CoV-2 target is the non-structural protein 16 (nsp16). Nsp16 forms a complex with another protein, nsp10, which is resposible for the methylation of the viral RNA ribose at the 2′-*O* position. This modification helps to hide the viral RNA from the host immunity, with the methyl donor being *S*-adenosyl-L-methionine (SAM) [77]. A crystal structure of SARS-CoV-2 nsp16 complexed with nsp10 and co-crystallized with SAM is available at the protein data bank (6W4H pdb code). The authors’ group investigated the potential affinity of the tested compounds towards the nsp16/10 complex. Initially, SAM was redocked in the active site to validate the docking procedure and the correct pose was detected (first pose with −8.1 kcal/mol energy). Among the tested compounds, **18**, **103**, **98**, **104**, and **124** attained docking energies better than that of SAM.

**Figure 21:**
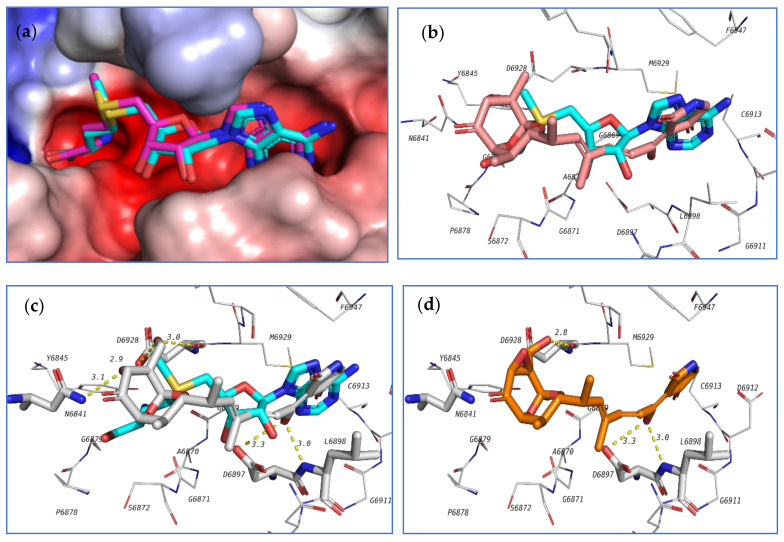
Docking results of the investigated compounds in the active site of SARS-CoV-2 methyltransferase (6W4H). (**a**) Validation of the docking procedure showing good matching between crystallized (blue) and docked (pink) ligands. (**b**) Docking pose and interactions of **103** (brick-red). (**c**) Docking pose and interactions of **98** (white). (**d**) Docking pose and interactions of **104** (orange).

The top 3 compounds **103**, **98**, and **104** are closely similar in structure and their binding poses were also found to be very similar, which suggested the selective recognition of nsp16/10 towards this class of compounds. The docking pose of this compound in the active site of nsp16/10 is shown in Figure 21.

The third investigated target is the SARS-CoV-2 RNA dependent RNA polymerase (RdRp, nsp12). This enzyme, in a complex form with nsp7 and nsp8, plays a key role in the replication of the positive strand viral RNA. Remdesivir in its triphosphate form is a known inhibitor to this class of enzymes [78]. Nsp12, complexed with nsp7, nsp8, remdesivir triphosphate and a short RNA sequence have been crystallized (7BV2 pdb code). This crystal structure was used to study the ability of the tested compounds to be docked in the active site of RdRp, which is the same position occupied by remdesivir triphosphate. Redocking of remdesivir triphosphate resulted in a similar pose to the crystal structure and attained an energy score of −7.0 kcal/mol.

**Figure 22:**
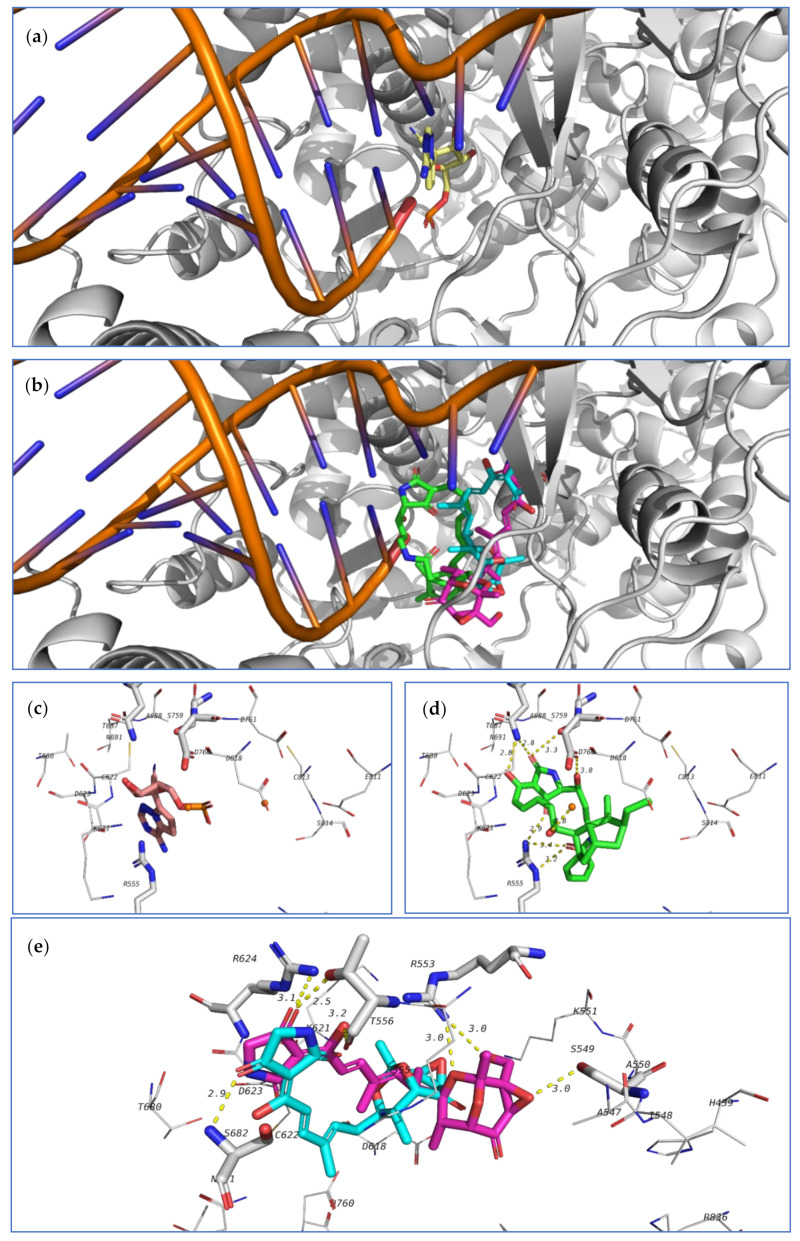
Docking results of the tested compounds in the active site of SARS-CoV-2 RdRp (7BV2). (**a**) Binding position of remdesivir. (**b**) Binding position of the docked ligands including **124** (green), **103** (blue), and **104** (pink). (**c**) Interactions between co-crystallized ligand with amino acids in the active site of RdRp. (**d**) Interactions of **124** (green). (**e**) Interactions of **103** (blue) and **104** (pink).

The co-crystallized ligand as well as the highest-docked compounds **124**, **103**, and **104** were docked in the same pocket which is formed at the interface between nsp12 and the RNA sequence as seen in Figure 22. Among these compounds, **124** exhibited noticeably good bonding energy (−9.0 kcal/mol) and several hydrogen bonds with the active site residues, which include T687, S759, D760, R555, and with Mg^2+^.

On the other hand, compounds **103** and **104** showed similar binding behavior, but flipped binding poses (Figure 22). The high stability of the complex of **124** formed in the active site of RdRp, and the formation of several other hydrogen bonds suggest that this compound might also be a good lead as an inhibitor of SARS-CoV-2 RdRp and should be further subjected to future investigation.

In addition to the above-mentioned targets, the authors’ group also studied the docking behavior of the investigated compounds in the active sites of both SARS-CoV-2 S-protein and human ACE2 receptors, which are involved in the viral recognition by human cells prior to infection, but none of them obtained a good docking energy.

## 7. Conclusions

Scleractiniae and their associated microorganisms have so far been largely studied as a promising source of metabolites with varied new chemical structures displaying a variety of interesting biological activities. These compounds present an enormous potential for the discovery of new therapeutic leads for drug development to fight the current global health problems with increasing numbers over the past years (Figure 1). Interestingly, many of these secondary metabolites show potent cytotoxic, anticancer, or antibacterial activities (Figure 7), with the major activity being cytotoxic. A docking study of selected compounds based on their physicochemical properties was done to investigate potential effects on SARS-CoV-2 targets. Among them, the tirandamycins **98**, **103,** and **104** showed potential inhibition against SARS-CoV-2 methyltransferase nsp16/10 suggesting that such class can be a good start for the design of SARS-CoV-2 inhibitors. In addition, alteramide A (**124**) has shown promising results against RdRp, which further implicate the importance of natural products repository as a source of new leads for the design of inhibitors against SARS-CoV-2. Scientific efforts should be continued for the purpose of exploring the stony coral environment in the search for new bioactive compounds for the development of new drugs getting benefit of the rising development of oceanographic science and metabolome screening techniques.

## Supplementary Materials

The following are available online at https://www.mdpi.com/1660-3397/18/12/645/s1, Table S1: Marine natural products isolated from Scleractinia-associated organisms; Table S2: Docking energies (kcal/mol) of investigated compounds with different SARS-CoV-2 targets.
