# Supplementary material for: Bioactivity Potential of Marine Natural Products from Scleractinia-Associated Microbes and In Silico Anti-SARS-COV-2 Evaluation
Source: Mar Drugs. 2020 Dec 16;18(12):645. doi: 10.3390/md18120645 (PMC7765564; doi:10.3390/md18120645)
Supplement: Supplementary file 1 [file marinedrugs-18-00645-s001.pdf]

Bioactivity Potential of Marine Natural Products from Scleractinia-Associated Microbes and *In Silico* Anti-SARS-COV-2 Evaluation

Eman Maher Zahran [1,#], Amgad Albohy [2,#], Amira Khalil [2], Alyaa Hatem Ibrahim [3], Heba Ali Ahmed [1], Ebaa M. El-Hossary [4], Gerhard Bringmann [5,*] and Usama Ramadan Abdelmohsen [1,6,*]

[1] Department of Pharmacognosy, Faculty of Pharmacy, Deraya University, Universities Zone, 61111 New Minia City, Egypt
[2] Department of Pharmaceutical Chemistry, Faculty of Pharmacy, The British University in Egypt (BUE), El-Sherouk City, Cairo-Suez Desert Road, 11837, Egypt
[3] Department of Pharmacognosy, Faculty of Pharmacy, Sohag University, 82524 Sohag, Egypt
[4] National Centre for Radiation Research & Technology, Egyptian Atomic Energy Authority, Ahmed El-Zomor St. 3, El-Zohoor Dist., Nasr City, 11765 Cairo, Egypt
[5] Institute of Organic Chemistry, University of Würzburg, Am Hubland, 97074 Würzburg, Germany
[6] Department of Pharmacognosy, Faculty of Pharmacy, Minia University, 61519 Minia, Egypt

* Correspondence: Gerhard Bringmann (bringman@chemie.uni-wuerzburg.de) and Usama Ramadan Abdelmohsen (usama.ramadan@mu.edu.eg)

\# These authors have equally contributed to this work

Table S1: Marine natural products isolated from Scleractinia-associated organisms

No.	Name	Chemical category	Source organism	Host	Biological activity	Geographical location	Reference
**1**	12-Dimethoxy-pinselin	Xanthone	*Scopulariopsis* sp.	*Stylophora* sp.	Not mentioned	Red sea, Egypt, Africa	[1]
**2**	12-*O*-acetyl-AGI-B4	Xanthone	*Scopulariopsis* sp.	*Stylophora* sp.	Not mentioned	Red sea, Egypt, Africa	[1]
**3**	AGI-B4	Xanthone	*Scopulariopsis* sp.	*Stylophora* sp.	Cytotoxic	Red sea, Egypt, Africa	[1]
**4**	Hyperxanthone C	Xanthone	*Scopulariopsis* sp.	*Stylophora* sp.	Not mentioned	Red sea, Egypt, Africa	[1]
**5**	Pinselin	Xanthone	*Scopulariopsis* sp.	*Stylophora* sp.	Immuno-suppressive	Red sea, Egypt, Africa	[1,2]
**6**	Sydowinin B	Xanthone	*Scopulariopsis* sp.	*Stylophora* sp.	Immuno-suppressive	Red sea, Egypt, Africa	[1,3]
**7**	13-*O*-acetyl-sydowinin B	Xanthone	*Scopulariopsis* sp.	*Stylophora* sp.	Antioxidant	Red sea, Egypt, Africa	[1,4]
**8**	2,11-Dihydroxy-1-methoxycarbonyl-9-carboxyl-xanthone	Xanthone	*Scopulariopsis* sp.	*Stylophora* sp.	Not mentioned	Red sea, Egypt, Africa	[1]
**9**	Sydowinin A	Xanthone	*Scopulariopsis* sp.	*Stylophora* sp.	Immuno-suppressive	Red sea, Egypt, Africa	[1,3]

**10**	8-(Methoxy-carbonyl)-1-hydroxy-9-oxo- 9H-xanthene-3-carboxylic acid	Xanthone	*Scopulariopsis* sp.	*Stylophora* sp.	Not mentioned	Red sea, Egypt, Africa	[1]
**11**	Methyl-3,8-dihydroxy-6-methyl-9-oxo-9H- xanthene-1-carboxylate	Xanthone	*Scopulariopsis* sp.	*Stylophora* sp.	Cytotoxic	Red sea, Egypt, Africa	[1,5]
**12**	Sydowic acid	Sesquiterpene	*Scopulariopsis* sp.	*Stylophora* sp.	Anti-phyto-pathogenic	Red sea, Egypt, Africa	[1,6]
**13**	Sydonic acid	Sesquiterpene	*Scopulariopsis* sp.	*Stylophora* sp.	Anti-phyto-pathogenic	Red sea, Egypt, Africa	[1,6]
**14**	11-Hydroxysydonic acid	Sesquiterpene	*Scopulariopsis* sp.	*Stylophora* sp.	Not mentioned	Red sea, Egypt, Africa	[1]
**15**	11,12-Dihydroxysydonic acid	Sesquiterpene	*Scopulariopsis* sp.	*Stylophora* sp.	Not mentioned	Red sea, Egypt, Africa	[1]
**16**	1-Hydroxy-boivinianic acid	Sesquiterpene	*Scopulariopsis* sp.	*Stylophora* sp.	Not mentioned	Red sea, Egypt, Africa	[1]
**17**	Violaceol I	Phenyl ether derivative	*Scopulariopsis* sp.	*Stylophora* sp.	Cytotoxic and antioxidant	Red sea, Egypt, Africa	[1,4]
**18**	Violaceol II	Phenyl ether derivative	*Scopulariopsis* sp.	*Stylophora* sp.	Cytotoxic and antioxidant	Red sea, Egypt, Africa	[1,4]
**19**	Diorcinol	Phenyl ether derivative	*Scopulariopsis* sp.	*Stylophora* sp.	Antioxidant	Red sea, Egypt, Africa	[1,4]

**20**	Rikuzenol	Phenyl ether derivative	*Scopulariopsis* sp.	*Stylophora* sp.	Not mentioned	Red sea, Egypt, Africa	[1]
**21**	Scopulamide	Alkaloid	*Scopulariopsis* sp.	*Stylophora* sp.	Not mentioned	Red sea, Egypt, Africa	[1]
**22**	Lumichrome	Alkaloid	*Scopulariopsis* sp.	*Stylophora* sp.	Not mentioned	Red sea, Egypt, Africa	[1]
**23**	WIN 64821	Alkaloid	*Scopulariopsis* sp.	*Stylophora* sp.	Not mentioned	Red sea, Egypt, Africa	[1]
**24**	Scopularide A	Cyclodepsi-peptide	*Scopulariopsis* sp.	*Stylophora* sp.	Cytotoxic	Red sea, Egypt, Africa	[1]
**25**	Scopularide B	Cyclodepsi-peptide	*Scopulariopsis* Sp.	*Stylophora* Sp.	Anticancer	Red sea, Egypt, Africa	[1,7]
**26**	Scopupyrone	α-Pyrone derivative	*Scopulariopsis* sp.	*Stylophora* sp.	Not mentioned	Red sea, Egypt, Africa	[1]
**27**	Pyrenochaetic acid	Benzoic acid derivative	*Scopulariopsis* sp.	*Stylophora* sp.	Cytotoxic	Red sea, Egypt, Africa	[1,8]
**28**	7-OH-2,5-Dimethyl-chromone	Chromone derivative	*Scopulariopsis* sp.	*Stylophora* sp.	Not mentioned	Red sea, Egypt, Africa	[1]
**29**	Ergosterol	Sterol	*Scopulariopsis* sp.	*Stylophora* sp.	Not mentioned	Red sea, Egypt, Africa	[1]
**30**	4-Methyl-candidusin A	Candidusin derivative	*Aspergillus tritici* SP2-8-1	*Galaxea fascicularis*	Cytotoxic Antibacterial	Malaysia, Asia	[9]
**31**	Aspetritone A	Anthraquinone derivative	*Aspergillus tritici* SP2-8-1	*Galaxea fascicularis*	Cytotoxic Antibacterial	Malaysia, Asia	[9]

**32**	Aspetritone B	Anthraquinone derivative	*Aspergillus tritici* SP2-8-1	*Galaxea fascicularis*	Cytotoxic Antibacterial	Malaysia, Asia	[9]
**33**	3,4-Dimethyl-3''-prenylcandidusin A	Prenylcandidusin derivative	*Aspergillus tritici* SP2-8-1	*Galaxea fascicularis*	Cytotoxic Antibacterial	Malaysia, Asia	[9]
**34**	4-Methyl-3''-prenylcandidusin A	Prenylcandidusin derivative	*Aspergillus tritici* SP2-8-1	*Galaxea fascicularis*	Cytotoxic Antibacterial	Malaysia, Asia	[9]
**35**	3,4-Dimethylcandidusin A	Candidusin derivative	*Aspergillus tritici* SP2-8-1	*Galaxea fascicularis*	Not mentioned	Malaysia, Asia	[9]
**36**	Candidusin A	Candidusin derivative	*Aspergillus tritici* SP2-8-1	*Galaxea fascicularis*	Cytotoxic and antibacterial	Malaysia, Asia	[9]
**37**	4,4'-Deoxyterphenyllin	Terphenyllin derivative	*Aspergillus tritici* SP2-8-1	*Galaxea fascicularis*	Not mentioned	Malaysia, Asia	[9]
**38**	4''-Deoxyterphenyllin	Terphenyllin derivative	*Aspergillus tritici* SP2-8-1	*Galaxea fascicularis*	Cytotoxic Antibacterial	Malaysia, Asia	[9]
**39**	3-Prenylterphenyllin	Terphenyllin derivative	*Aspergillus tritici* SP2-8-1	*Galaxea fascicularis*	Cytotoxic Antibacterial	Malaysia, Asia	[9]
**40**	Terphenyllin	Terphenyllin derivative	*Aspergillus tritici* SP2-8-1	*Galaxea fascicularis*	Cytotoxic Antibacterial	Malaysia, Asia	[9]
**41**	3-Hydroxyterphenyllin	Terphenyllin derivative	*Aspergillus tritici* SP2-8-1	*Galaxea fascicularis*	Cytotoxic Antibacterial	Malaysia, Asia	[9]

**42**	3-Hydroxy-3''-deoxyterphenyllin	Terphenyllin derivative	*Aspergillus tritici* SP2-8-1	*Galaxea fascicularis*	Not mentioned	Malaysia, Asia	[9]
**43**	3''-Prenylterphenyllin	Terphenyllin derivative	*Aspergillus tritici* SP2-8-1	*Galaxea fascicularis*	Cytotoxic Antibacterial	Malaysia, Asia	[9]
**44**	Emodin	Anthraquinone derivative	*Aspergillus tritici* SP2-8-1	*Galaxea fascicularis*	Cytotoxic Antibacterial	Malaysia, Asia	[9]
**45**	3-Hydroxy- 1,2,5,6-tetramethoxyanthracene-9,10-dione	Anthraquinone derivative	*Aspergillus tritici* SP2-8-1	*Galaxea fascicularis*	Cytotoxic Antibacterial	Malaysia, Asia	[9]
**46**	3-Hydroxy-2-hydroxymethyl-1-methoxyanthracene-9,10-dione	Anthraquinone derivative	*Aspergillus tritici* SP2-8-1	*Galaxea fascicularis*	Not mentioned	Malaysia, Asia	[9]
**47**	1,2,3-Trimethoxy-7-hydroxymethylanthracene-9,10-dione	Anthraquinone derivative	*Aspergillus tritici* SP2-8-1	*Galaxea fascicularis*	Not mentioned	Malaysia, Asia	[9]
**48**	3β,7β,15α,24-Tetrahydroxyolean-12-ene-11,22-dione	Triterpene	*Scopulariopsis* sp.	*Stylophora* sp.	Not mentioned	Red Sea, Egypt, Africa	[10]
**49**	15α,22β,24-Trihydroxyolean-11,13-diene-3-one	Triterpene	*Scopulariopsis* sp.	*Stylophora* sp.	Not mentioned	Red Sea, Egypt, Africa	[10]
**50**	7β,15α,24-Trihydroxyolean-12-ene-3,11,22-trione	Triterpene	*Scopulariopsis* sp.	*Stylophora* sp.	Not mentioned	Red Sea, Egypt, Africa	[10]

**51**	15α,24-Dihydroxyolean-12-ene-3,11,22-trione	Triterpene	*Scopulariopsis* sp.	*Stylophora* sp.	Not mentioned	Red Sea, Egypt, Africa	[10]
**52**	Soyasapogenol B	Triterpene	*Scopulariopsis* sp.	*Stylophora* sp.	Antimutagenic, antiviral and anti-inflammatory	Red Sea, Egypt, Africa	[10,11]
**53**	(2*E*, 4*E*)-4′-Dihydrophaseic acid	Sesquiterpene	*Scopulariopsis* sp.	*Stylophora* sp.	Not mentioned	Red Sea, Egypt, Africa	[10]
**54**	2*Z*, 4*E*)-4′-Dihydrophaseic acid	Sesquiterpene	*Scopulariopsis* sp.	*Stylophora* sp.	Not mentioned	Red Sea, Egypt, Africa	[10]
**55**	6-Hydroxy-2,7-dimethyl-1,4-naphthoquinone	Polyketide	*Scopulariopsis* sp.	*Stylophora* sp.	Not mentioned	Red Sea, Egypt, Africa	[10]
**56**	6-Hydroxy-2,2-dimethyl-2*H*-chromene	Polyketide	*Scopulariopsis* sp.	*Stylophora* sp.	Antioxidant and cancer protecting effects	Red Sea, Egypt, Africa	[10,12]
**57**	Scoparone	Polyketide	*Scopulariopsis* sp.	*Stylophora* sp.	Immuno-suppressive, antiproliferative and antioxidant	Red Sea, Egypt, Africa	[10,13-15]
**58**	5-Methyluracil	Nitrogenous compound	*Scopulariopsis* sp.	*Stylophora* sp.	Antitumor	Red Sea, Egypt, Africa	[10,16]

**59**	4-Hydroxy-3-methoxy-2(1*H*)-quinolinone	Nitrogenous compound	*Scopulariopsis* sp.	*Stylophora* sp.	Not mentioned	Red Sea, Egypt, Africa	[10]
**60**	4-Hydroxyphenyl-glyoxylic acid amide	Nitrogenous compound	*Scopulariopsis* sp.	*Stylophora* sp.	Not mentioned	Red Sea, Egypt, Africa	[10]
**61**	Indole-3-carboxaldehyde	Nitrogenous compound	*Scopulariopsis* sp.	*Stylophora* sp.	Not mentioned	Red Sea, Egypt, Africa	[10]
**62**	Indole-3-carboxylic acid	Nitrogenous compound	*Scopulariopsis* sp.	*Stylophora* sp.	Not mentioned	Red Sea, Egypt, Africa	[10]
**63**	(1*H*-Indol-3-yl)-oxo-acetamide	Nitrogenous compound	*Scopulariopsis* sp.	*Stylophora* sp.	Not mentioned	Red Sea, Egypt, Africa	[10]
**64**	*N*-Acetyl-β-oxo-tryptamine	Nitrogenous compound	*Scopulariopsis* sp.	*Stylophora* sp.	Not mentioned	Red Sea, Egypt, Africa	[10]
**65**	Gliomastin A	Hydroquinone derivative	*Gliomastix* sp.	*Stylophora* sp.	Cytotoxic	Red Sea, Egypt, Africa	[17]
**66**	Gliomastin B	Hydroquinone derivative	*Gliomastix* sp.	*Stylophora* sp.	Not mentioned	Red Sea, Egypt, Africa	[17]
**67**	Gliomastin C	Hydroquinone derivative	*Gliomastix* sp.	*Stylophora* sp.	Antitubercular	Red Sea, Egypt, Africa	[17]
**68**	Gliomastin D	Hydroquinone derivative	*Gliomastix* sp.	*Stylophora* sp.	Not mentioned	Red Sea, Egypt, Africa	[17]
**69**	9-*O*-Methyl-gliomastin C	Hydroquinone derivative	*Gliomastix* sp.	*Stylophora* sp.	Not mentioned	Red Sea, Egypt, Africa	[17]

**70**	Acremonin A 1-*O*-β-D-gluco- pyranoside	Hydroquinone derivative	*Gliomastix* sp.	*Stylophora* sp.	Not mentioned	Red Sea, Egypt, Africa	[17]
**71**	Gliomastin E 1-*O*- β-D-gluco -pyranoside	Hydroquinone derivative	*Gliomastix* sp.	*Stylophora* sp.	Not mentioned	Red Sea, Egypt, Africa	[17]
**72**	6′-*O*-Acetyl-isohomoarbutin	Hydroquinone derivative	*Gliomastix* sp.	*Stylophora* sp.	Not mentioned	Red Sea, Egypt, Africa	[17]
**73**	Isohomoarbutin	Hydroquinone derivative	*Gliomastix* sp.	*Stylophora* sp.	Not mentioned	Red Sea, Egypt, Africa	[17]
**74**	2-Methyl-1,4-benzenediol	Hydroquinone derivative	*Gliomastix* sp.	*Stylophora* sp.	Cytotoxic Antitubercular Antibacterial	Red Sea, Egypt, Africa	[17]
**75**	Acremonin A	Hydroquinone derivative	*Gliomastix* sp.	*Stylophora* sp.	Cytotoxic Antitubercular	Red Sea, Egypt, Africa	[17]
**76**	Prenylhydro-quinone	Hydroquinone derivative	*Gliomastix* sp.	*Stylophora* sp.	Cytotoxic Antitubercular Antibacterial	Red Sea, Egypt, Africa	[17]
**77**	F-11334A_1	Hydroquinone derivative	*Gliomastix* sp.	*Stylophora* sp.	Cytotoxic Antitubercular	Red Sea, Egypt, Africa	[17]
**78**	(R)-2-(2-Hydroxy-propan2-yl)-2,3-dihydro-5-hydroxybenzofuran	Hydroquinone derivative	*Gliomastix* sp.	*Stylophora* sp.	Not mentioned	Red Sea, Egypt, Africa	[17]
**79**	2,2-Dimethyl-chroman-3,6-diol	Hydroquinone derivative	*Gliomastix* sp.	*Stylophora* sp.	Not mentioned	Red Sea, Egypt, Africa	[17]

**80**	Verruculosin A	Oligophen-alenone dimer	*Talaromyces verruculosus*	*Goniopora* sp.	Antitumor	South China Sea, China, Asia	[18]
**81**	Verruculosin B	Oligophen-alenone dimer	*Talaromyces verruculosus*	*Goniopora* sp.	Antitumor	South China Sea, China, Asia	[18]
**82**	Bacillisporin F	Oligophen-alenone dimer	*Talaromyces verruculosus*	*Goniopora* sp.	Antitumor	South China Sea, China, Asia	[18]
**83**	Duclauxin	Oligophen-alenone dimer	*Talaromyces verruculosus*	*Goniopora* sp.	Antitumor and antileukemia	South China Sea, China, Asia	[18]
**84**	Xenoclauxin	Oligophen-alenone dimers	*Talaromyces verruculosus*	*Goniopora* sp.	Antitumor	South China Sea, China, Asia	[18]
**85**	Erythroxanthin sulfate	Carotenoid	*Erythrobacter flavus*	*Acropora nasuta*	Not mentioned	Indonesia	[19]
**86**	Ketonostoxanthin	Carotenoid	*Erythrobacter flavus*	*Acropora nasuta*	Not mentioned	Indonesia	[19]
**87**	Nostoxanthin sulfate	Carotenoid	*Erythrobacter flavus*	*Acropora nasuta*	Not mentioned	Indonesia	[19]
**88**	Caloxanthin sulfate	Carotenoid	*Erythrobacter flavus*	*Acropora nasuta*	Not mentioned	Indonesia	[19]
**89**	Nostoxanthin	Carotenoid	*Erythrobacter flavus*	*Acropora nasuta*	Not mentioned	Indonesia	[19]
**90**	Zeaxanthin sulfate	Carotenoid	*Erythrobacter flavus*	*Acropora nasuta*	Not mentioned	Indonesia	[19]
**91**	Caloxanthin	Carotenoid	*Erythrobacter flavus*	*Acropora nasuta*	Not mentioned	Indonesia	[19]
**92**	Bacterio-rubixanthinal	Carotenoid	*Erythrobacter flavus*	*Acropora nasuta*	Not mentioned	Indonesia	[19]
**93**	Zeaxanthin	Carotenoid	*Erythrobacter flavus*	*Acropora nasuta*	Radioprotectant and antioxidant	Indonesia	[19-21]

**94**	β-Cryptoxanthin	Carotenoid	*Erythrobacter flavus*	*Acropora nasuta*	Antioxidant, anti-inflammatory, anticancer, homeostatic and anti-osteoporosis	Indonesia	[19,22]
**95**	Bacteriochlorophyll	Carotenoid	*Erythrobacter flavus*	*Acropora nasuta*	Not mentioned	Indonesia	[19]
**96**	β-Carotene	Carotenoid	*Erythrobacter flavus*	*Acropora nasuta*	Antioxidant and anticancer	Indonesia	[19,23]
**97**	Aranciamycin K	Anthracycline analogue	*Streptomyces* sp.	*Porites* sp.	Not mentioned	China	[24]
**98**	Isotirandamycin B	Tirandamycin analogue	*Streptomyces* sp.	*Porites* sp.	Bacteriostatic	China	[24]
**99**	γ-Rhodomycinone	Anthracycline derivative	*Streptomyces* sp.	*Porites* sp.	Not mentioned	China	[24]
**100**	β-Rhodomycinone	Anthracycline derivative	*Streptomyces* sp.	*Porites* sp.	Cytotoxic	China	[24]
**101**	262-6	Anthracycline derivative	*Streptomyces* sp.	*Porites* sp.	Antibacterial Cytotoxic	China	[24]
**102**	β-Rhodomycin-II	Anthracycline derivative	*Streptomyces* sp.	*Porites* sp.	Antibacterial Cytotoxic	China	[24]
**103**	Tirandamycin A	Tirandamycin derivative	*Streptomyces* sp.	*Porites* sp.	Antibacterial	China	[24]

**104**	Tirandamycin B	Tirandamycin derivative	*Streptomyces* sp.	*Porites* sp.	Antibacterial	China	[24]
**105**	Pelopuradazole	Imidazole alkaloid	*Pelomonas puraquae*	*Acropora* sp.	Not mentioned	South China Sea	[25]
**106**	3*H*-Imidazole-4-carboxylic acid	Imidazole alkaloid	*Pelomonas puraquae*	*Acropora* sp.	Not mentioned	South China Sea	[25]
**107**	2-Methyl-3H-imidazole-4-carboxylic acid	Imidazole alkaloid	*Pelomonas puraquae*	*Acropora* sp.	Not mentioned	South China Sea	[25]
**108**	1*H*-Pyrrole-2-carboxylic acid	Imidazole alkaloid	*Pelomonas puraquae*	*Acropora* sp.	Antibacterial	South China Sea	[25]
**109**	Pelopurin A	Cyclodipeptide	*Pelomonas puraquae*	*Acropora* sp.	Not mentioned	South China Sea	[25]
**110**	Pelopurin B	Cyclodipeptide	*Pelomonas puraquae*	*Acropora* sp.	Not mentioned	South China Sea	[25]
**111**	Unnamed	Hydroxy-quinaldic acid derivative	*Streptomyceces cyaneofuscatus*	Different corals belong to *Scleractinia*	Cytotoxic	Northeast Atlantic, Europe	[26]
**112**	Unnamed	Hydroxy-quinaldic acid derivative	*Streptomyceces cyaneofuscatus*	Different corals belong to *Scleractinia*	Not mentioned	Northeast Atlantic, Europe	[26]
**113**	Unnamed	Hydroxy-quinaldic acid derivative	*Streptomyceces cyaneofuscatus*	Different corals belong to *Scleractinia*	Not mentioned	Northeast Atlantic, Europe	[26]
**114**	3-Hydroxyquinaldic acid	Hydroxyquinaldic acid derivative	*Streptomyceces cyaneofuscatus*	Different corals belong to *Scleractinia*	Not mentioned	Northeast Atlantic, Europe	[26]

**115**	3- Hydroxyquinaldic acid amide	Hydroxyquinaldic acid derivative	*Streptomyceces cyaneofuscatus*	Different corals belong to *Scleractinia*	Cytotoxic	Northeast Atlantic, Europe	[26]
**116**	Nakienone A	Cyclic C-11	*Synechocystis* sp.	*Acropora* sp.	Cytotoxic	Okinawa, Japan, Asia	[27]
**117**	Nakitriol	Cyclic C-11	*Synechocystis* sp.	*Acropora* sp.	Cytotoxic	Okinawa, Japan, Asia	[27]
**118**	Nakienone B	Cyclic C-11	*Synechocystis* sp.	*Acropora* sp.	Not mentioned	Okinawa, Japan, Asia	[27]
**119**	Nakienone C	Cyclic C-11	*Synechocystis* sp.	*Acropora* sp.	Not mentioned	Okinawa, Japan, Asia	[27]
**120**	Pitiamide A	Fatty acid amide	*Phormidium corallyticum*	*Porites* sp.	Anti-proliferative	USA, North America	[28]
**121**	1*E*-Pitiamide B	Fatty acid amide	*Phormidium corallyticum*	*Porites* sp.	Anti-proliferative	USA, North America	[28]
**122**	Looekeyolide A	Macrolide	*Roseofilum reptotaenium*	*Montastraea* sp.	Not mentioned	Florida, North America	[29]
**123**	Looekeyolide B	Macrolide	*Roseofilum reptotaenium*	*Montastraea* sp.	Not mentioned	Florida, North America	[29]
**124**	Alteramide A	Tetracyclic alkaloid	*Pseudoalteromonas* sp.	*Montipora* sp.	Cytotoxic and antifungal	Japan, Asia	[30,31]
**125**	Alteramide B	Tetracyclic alkaloid	*Pseudoalteromonas* sp.	*Montipora* sp.	Antifungal	Japan, Asia	[30,31]
**126**	(2*Z*,4*E*)-3-Methyl-2,4-decadienoic acid	Fatty acid	*Microbulbifer* sp.	*Porites* sp.	Antibacterial	Japan, Asia	[32]

**127**	Nesteretal A	Polyhemiketal,	*Nesterenkonia* sp.	*Platygyra* sp.	RXRα activity	China Asia	[33]
**128**	Lobophorin K	Lobophorin	*Streptomyces* sp.	*Lophelia pertusa*	Cytotoxic Antibacterial	Cantabrian Sea, North America	[34]
**129**	Gorgosterol	Sterol	*Zooxanthellae*	*Oculina diffusa*	Not mentioned	Permuda, Europe	[35]
**130**	23-Desmethyl-gorgosterol	Sterol	*Zooxanthellae*	*Oculina diffusa*	Not mentioned	Permuda, Europe	[35]
**131**	Dinosterol	Sterol	*Zooxanthellae*	*Oculina diffusa*	Not mentioned	Permuda, Europe	[35]
**132**	Cholesterol	Sterol	*Zooxanthellae*	*Oculina diffusa*	Not mentioned	Permuda, Europe	[35]
**133**	4α-(24S)-Dimethyl-cholesta-3β-ol	Sterol	*Zooxanthellae*	*Oculina diffusa*	Not mentioned	Permuda, Europe	[35]
**134**	4α-(24R)-Dimethyl-cholesta-22-en-3β-ol	Sterol	*Zooxanthellae*	*Oculina diffusa*	Not mentioned	Permuda, Europe	[35]

Table S2: Docking energies (kcal/mol) of investigated compounds with different SARS-CoV-2 targets

Compound Number	6LU7	6W4H, nsp16	7BV2, nsp12	6VW1	6M0J
	Main protease, M^{pro}	Methyl Transferase	RNA-dependent RNA polymerase	Human ACE2	Receptor-binding domain (RBD) of S-protein
**3**	-7.4	-7.1	-6.3	-5.6	-6.1
**7**	-6.9	-7.9	-7	-6.4	-6.4
**17**	-6.6	-7.6	-6.2	-5.8	-6.4
**18**	-7.7	-8.2	-6.3	-7	-6.7
**31**	-7.1	-7.3	-6.3	-5.2	-5.6
**32**	-7.4	-7.2	-6.3	-5.9	-5.9
**39**	-7.9	-8.0	-6.3	-5.8	-6.7
**53**	-6.1	-6.3	-5.9	-6.1	-5.9
**77**	-5.8	-6.5	-5.4	-5.3	-5.6
**98**	-7.8	-8.4	-7.6	-5.9	-6.8
**103**	-7.9	-8.5	-8.1	-6.3	-6.8
**104**	-7.8	-8.3	-7.9	-5.9	-6.8
**120**	-5.8	-5.8	-5	-5	-5.1
**121**	-5.2	-6.3	-4.8	-5.2	-4.8
**124**	-7.1	-8.2	-9	-6.1	-7.4
**Ligand**	-7.9 (N3)	-8.1 (SAM)	-7.0 (RemdesivirTP)	----	---

References

1. Elnaggar, M.S.; Ebada, S.S.; Ashour, M.L.; Ebrahim, W.; Müller, W.E.; Mándi, A.; Kurtán, T.; Singab, A.; Lin, W.; Liu, Z. Xanthones and sesquiterpene derivatives from a marine-derived fungus *Scopulariopsis* sp. *Tetrahedron* **2016**, *72*, 2411-2419.
2. Fujimoto, H.; Asai, T.; Kim, Y.-P.; Ishibashi, M. Nine Constituents Including Six Xanthone-Related Compounds Isolated from Two Ascomycetes, *Gelasinospora santi-florii* and *Emericella quadrilineata*, Found in a Screening Study Focused on Immunomodulatory Activity. *Chem. Pharm. Bull.* **2006**, *54*, 550-553.
3. Liu, H.; Chen, S.; Liu, W.; Liu, Y.; Huang, X.; She, Z. Polyketides with Immunosuppressive Activities from Mangrove Endophytic Fungus *Penicillium* sp. ZJ-SY_2. *Mar. Drugs* **2016**, *14*, 217.
4. Liu, S.; Wang, H.; Su, M.; Hwang, G.J.; Hong, J.; Jung, J.H. New metabolites from the sponge-derived fungus *Aspergillus sydowii* J05B-7F-4. *Nat. Prod. Res.* **2017**, *31*, 1682-1686.
5. Dalinova, A.; Chisty, L.; Kochura, D.; Garnyuk, V.; Petrova, M.; Prokofieva, D.; Yurchenko, A.; Dubovik, V.; Ivanov, A.; Smirnov, S. Isolation and Bioactivity of Secondary Metabolites from Solid Culture of the Fungus, *Alternaria sonchi*. *Biomolecules* **2020**, *10*, 81.
6. Bunbamrung, N.; Intaraudom, C.; Supothina, S.; Komwijit, S.; Pittayakhajonwut, P. Antibacterial and anti-phytopathogenic substances from the insect pathogenic fungus *Gibellula* sp. BCC36964. *Phytochem. Lett.* **2015**, *12*, 142-147.
7. Kramer, A.; Beck, H.C.; Kumar, A.; Kristensen, L.P.; Imhoff, J.F.; Labes, A. Proteomic Analysis of Anti-Cancerous Scopularide Production by a Marine *Microascus brevicaulis* Strain and Its UV Mutant. *PloS one* **2015**, *10*.
8. Zhang, H.; Mao, L.-L.; Qian, P.-T.; Shan, W.-G.; Wang, J.-D.; Bai, H. Two new metabolites from a soil fungus *Curvularia affinis* strain HS-FG-196. *J. Asian. Nat. Prod. Res.* **2012**, *14*, 1078-1083.
9. Wang, W.; Liao, Y.; Tang, C.; Huang, X.; Luo, Z.; Chen, J.; Cai, P. Cytotoxic and Antibacterial Compounds from the Coral-Derived Fungus *Aspergillus tritici* SP2-8-1. *Mar. drugs* **2017**, *15*, 348.
10. Elnaggar, M.S.; Ebada, S.S.; Ashour, M.L.; Ebrahim, W.; Singab, A.; Lin, W.; Liu, Z.; Proksch, P. Two new triterpenoids and a new naphthoquinone derivative isolated from a hard coral-derived fungus *Scopulariopsis* sp. *Fitoterapia* **2017**, *116*, 126-130.
11. Amin, H.; Abo Elsoud, M.; Sahab, A. Whole cell biocatalyst for soyasapogenol b production from soybean saponin. *The Open Conference Proceedings Journal* **2016**, *7*, 134-143.
12. Babu, K.S.; Raju, B.C.; Praveen, B.; Kishore, K.H.; Murty, U.S.; Rao, J.M. Microwave assisted synthesis and antimicrobial activity of 2, 2-dimethyl chromenes. *Heterocycl. Comm.* **2003**, *9*, 519-526.
13. Huei-Chen, H.; Shu-Hsun, C.; Chao, P.-D.L. Vasorelaxants from chinese herbs, emodin and scoparone, possess immunosuppressive properties. *Eur. J. Pharmacol.* **1991**, *198*, 211-213.
14. Park, S.; Kim, J.-K.; Oh, C.J.; Choi, S.H.; Jeon, J.-H.; Lee, I.-K. Scoparone interferes with STAT3-induced proliferation of vascular smooth muscle cells. *Exp. Mol. Med.* **2015**, *47*, e145-e145.
15. Atmaca, M.; Bilgin, H.M.; Obay, B.D.; Diken, H.; Kelle, M.; Kale, E. The hepatoprotective effect of coumarin and coumarin derivates on carbon tetrachloride-induced hepatic injury by antioxidative activities in rats. *J. Physiol. Biochem.* **2011**, *67*, 569.

16. Welchinska, E. Anti-tumor activity of bacterial lectin and 5-methyluracil adduct. *CBU International Conference Proceedings* **2014**, *2*, 307.

17. Elnaggar, M.S.; Ebrahim, W.; Mándi, A.; Kurtán, T.; Müller, W.E.; Kalscheuer, R.; Singab, A.; Lin, W.; Liu, Z.; Proksch, P. Hydroquinone derivatives from the marine-derived fungus *Gliomastix* sp. *RSC Adv.* **2017**, *7*, 30640-30649.

18. Wang, M.; Yang, L.; Feng, L.; Hu, F.; Zhang, F.; Ren, J.; Qiu, Y.; Wang, Z. Verruculosins A–B, New Oligophenalenone Dimers from the Soft Coral-Derived Fungus *Talaromyces verruculosus*. *Mar. drugs* **2019**, *17*, 516.

19. Setiyono, E.; Pringgenies, D.; Shioi, Y.; Kanesaki, Y.; Awai, K.; Brotosudarmo, T.H.P. Sulfur-Containing Carotenoids from A Marine Coral Symbiont *Erythrobacter flavus* Strain KJ5. *Mar. drugs* **2019**, *17*, 349.

20. Santocono, M.; Zurria, M.; Berrettini, M.; Fedeli, D.; Falcioni, G. Influence of astaxanthin, zeaxanthin and lutein on DNA damage and repair in UVA-irradiated cells. *J. Photochem. Photobiol. B, Biol.* **2006**, *85*, 205-215.

21. Havaux, M.; Dall'Osto, L.; Bassi, R. Zeaxanthin Has Enhanced Antioxidant Capacity with Respect to All Other Xanthophylls in Arabidopsis Leaves and Functions Independent of Binding to PSII Antennae. *Plant Physiol.* **2007**, *145*, 1506-1520.

22. Burri, B.J.; La Frano, M.R.; Zhu, C. Absorption, metabolism, and functions of β-cryptoxanthin. *Nutr. Rev.* **2016**, *74*, 69-82.

23. Bogacz-Radomska, L.; Harasym, J. Β-carotene—properties and production methods. *Food Quality and Safety* **2018**, *2*.

24. Cong, Z.; Huang, X.; Liu, Y.; Liu, Y.; Wang, P.; Liao, S.; Yang, B.; Zhou, X.; Huang, D.; Wang, J. Cytotoxic anthracycline and antibacterial tirandamycin analogues from a marine-derived *Streptomyces* sp. SCSIO 41399. *J. Antibiot.* **2019**, *72*, 45-49.

25. He, X.-X.; Chen, X.-J.; Peng, G.-T.; Guan, S.-Y.; Lei, L.-F.; Yao, J.-H.; Liu, B.-X.; Zhang, C.-X. Pelopuradazole, a new imidazole derivative alkaloid from the marine bacteria *Pelomonas puraquae* sp. nov.. *Nat. Prod. Res.* **2014**, *28*, 680-682.

26. Ortiz-López, F.J.; Alcalde, E.; Sarmiento-Vizcaíno, A.; Díaz, C.; Cautain, B.; García, L.A.; Blanco, G.; Reyes, F. New 3-Hydroxyquinaldic Acid Derivatives from Cultures of the Marine Derived Actinomycete *Streptomyces cyaneofuscatus* M-157. *Mar. drugs* **2018**, *16*, 371.

27. Nagle, D.G.; Gerwick, W.H. Nakienones A-C and nakitriol, new cytotoxic cyclic C_{11} metabolites from an okinawan cyanobacterial (*Synechocystis* sp.) overgrowth of coral. *Tetrahedron Lett.* **1995**, *36*, 849-852.

28. Cai, W.; Matthews, J.H.; Paul, V.J.; Luesch, H. Pitiamides a and b, multifunctional fatty acid amides from marine cyanobacteria. *Planta Med.* **2016**, *82*, 897-902.

29. Gunasekera, S.P.; Meyer, J.L.; Ding, Y.; Abboud, K.A.; Luo, D.; Campbell, J.E.; Angerhofer, A.; Goodsell, J.L.; Raymundo, L.J.; Liu, J. Chemical and Metagenomic Studies of the Lethal Black Band Disease of Corals Reveal Two Broadly Distributed, Redox-Sensitive Mixed Polyketide/Peptide Macrocycles. *J. Nat. Prod.* **2019**, *82*, 111-121.

30. Shigemori, H.; Bae, M.A.; Yazawa, K.; Sasaki, T.; Kobayashi, J. AAlteramide A, a new tetracyclic alkaloid from a bacterium *Alteromonas* sp. associated with the marine sponge *Halichondria okadai*. *J. Org. Chem.* **1992**, *57*, 4317-4320.

31. Moree, W.J.; McConnell, O.J.; Nguyen, D.D.; Sanchez, L.M.; Yang, Y.-L.; Zhao, X.; Liu, W.-T.; Boudreau, P.D.; Srinivasan, J.; Atencio, L., *et al.* Microbiota of healthy corals are active against fungi in a light-dependent manner. *ACS Chem. Biol.* **2014**, *9*, 2300-2308.

32. Sharma, A.R.; Harunari, E.; Zhou, T.; Trianto, A.; Igarashi, Y. I Isolation and biosynthesis of an unsaturated fatty acid with unusual methylation pattern from a coral-associated bacterium *Microbulbifer* sp. *Beilstein J. Org. Chem.* **2019**, *15*, 2327-2332.

33. Xie, C.-L.; Chen, R.; Yang, S.; Xia, J.-M.; Zhang, G.-Y.; Chen, C.-H.; Zhang, Y.; Yang, X.-W. Nesteretal A, A Novel Class of Cage-Like Polyketide from Marine-Derived Actinomycete *Nesterenkonia halobia*. *Org. Lett.* **2019**, *21*, 8174-8177.

34. Braña, A.F.; Sarmiento-Vizcaíno, A.; Osset, M.; Pérez-Victoria, I.; Martín, J.; De Pedro, N.; De la Cruz, M.; Díaz, C.; Vicente, F.; Reyes, F. Lobophorin K, a New Natural Product with Cytotoxic Activity Produced by *Streptomyces* sp. M-207 Associated with the Deep-Sea Coral *Lophelia pertusa*. *Mar. drugs* **2017**, *15*, 144.

35. Withers, N.W.; Kokke, W.; Fenical, W.; Djerassi, C. Sterol patterns of cultured zooxanthellae isolated from marine invertebrates: Synthesis of gorgosterol and 23-desmethylgorgosterol by aposymbiotic algae. *Proc. Natl. Acad. Sci. U.S.A.* **1982**, *79*, 3764-3768.
